# Ceramicines U–Z from *Chisocheton ceramicus* and structure–antimalarial activity relationship study

**DOI:** 10.1007/s11418-023-01746-2

**Published:** 2023-09-10

**Authors:** Alfarius Eko Nugroho, Tomoyuki Komuro, Takuya Kawaguchi, Yusuke Shindo, Chin Piow Wong, Yusuke Hirasawa, Toshio Kaneda, Takahiro Tougan, Toshihiro Horii, A. Hamid A. Hadi, Hiroshi Morita

**Affiliations:** 1 Faculty of Pharmaceutical Sciences, Hoshi University, Ebara 2-4-41 Shinagawa-Ku, Tokyo, 142-8501 Japan; 2 Research Center for Infectious Disease Control, Research Institute for Microbial Diseases, Osaka University, 3-1 Yamadaoka, Suita, Osaka 565-0871 Japan; 3 Department of Malaria Vaccine Development, Research Institute for Microbial Diseases, Osaka University, 3-1 Yamadaoka, Suita, Osaka 565-0871 Japan; 4 Department of Chemistry, Faculty of Science, University of Malaya, 50603 Kuala Lumpur, Malaysia

**Keywords:** Ceramicines U–Z, Limonoids, *Chisocheton ceramicus*, Meliaceae, Antimalarial activity, Structure–antimalarial activity relationship

## Abstract

Ceramicines are a series of limonoids which were isolated from the barks of Malaysian *Chisocheton ceramicus* (Meliaceae), and were known to show various biological activity. Six new limonoids, ceramicines U–Z (**1**–**6**), with a cyclopentanone[α]phenanthrene ring system with a β-furyl ring at C-17 were isolated from the barks of *C. ceramicus*. Their structures were determined on the basis of the 1D and 2D NMR analyses, and their absolute configurations were investigated by CD spectroscopy. Ceramicine W (**3**) exhibited potent antimalarial activity against *Plasmodium falciparum* 3D7 strain with IC_50_ value of 1.2 µM. In addition, the structure–antimalarial activity relationship (SAR) of the ceramicines was investigated to identify substituent patterns that may enhance activity. It appears that ring B and the functional groups in the vicinity of rings B and C are critical for the antimalarial activity of the ceramicines. In particular, bulky ester substituents with equatorial orientation at C-7 and C-12 greatly increase the antimalarial activity.

**Graphical Abstract:**

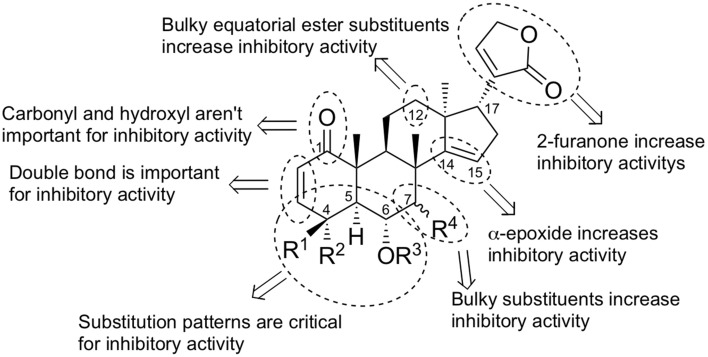

**Supplementary Information:**

The online version contains supplementary material available at 10.1007/s11418-023-01746-2.

## Introduction

Malaria is the largest parasitic protozoan infection in humankind in which malaria infects humans through the anopheles mosquito. Malaria is not only widespread throughout the tropics, but also occurs in many temperate regions. In light of this problem, scientists have turned to naturally occurring compounds obtained from plants used in the traditional medicine [1].

**Fig. 1:**
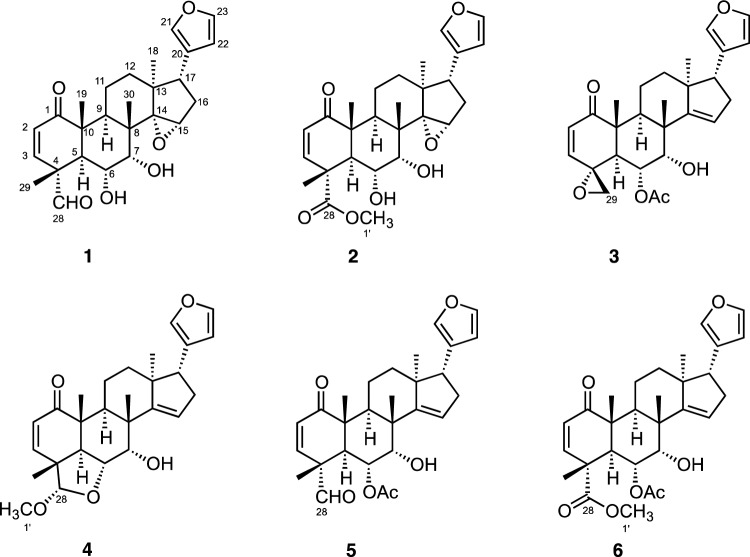
Structures of **1**–**6**

In our search for new bioactive compounds from medicinal plants, we have reported the isolation of numerous new compounds and their bioactivities, including alkaloids showing antimalarial and vasorelaxant activity [2–8], quassinoids [9, 10], and terpenoids [11–25]. In particular, we have found that *Chisocheton ceramicus* is rich in bioactive limonoids with antimalarial activity and have continuously investigated it. In this report, we describe the isolation, structure elucidation, and antimalarial activity of six new limonoids, ceramicines U–Z (**1**–**6**, Fig. 1). In addition, will also discuss the structure–antimalarial activity relationship of the ceramicines.

## Results and discussion

**Fig. 2:**
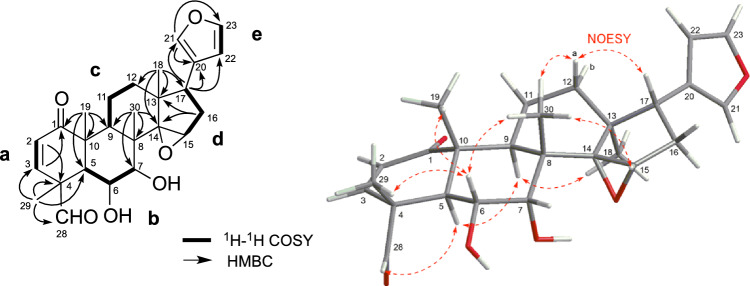
Selected 2D NMR correlations of **1**

Ceramicine U (**1**) was obtained as white amorphous solid, and the HRESIMS displayed a pseudomolecular ion peak at 463.2127 (M + Na)^+^ corresponding to the molecular formula C_26_H_32_O_6_. IR absorptions indicated the presence of hydroxy (3371 cm^−1^) and carbonyl (1721 and 1685 cm^−1^) groups. The ^1^H NMR spectrum (Table 1) suggested the presence of a furan (δ_H_ 6.24, 7.16, and 7.34), an aldehyde (δ_H_ 9.28) and 4 methyls (δ_H_ 0.84–1.54). The ^13^C NMR spectrum revealed 26 carbon resonances due to two carbonyls, one sp^2^ quaternary carbon, five sp^3^ quaternary carbons, five sp^2^ methines, five sp^3^ methines, three sp^3^ methylenes, and four methyls (Table 1). Overall, the ^1^H and ^13^C NMR data of **1** were similar to those of ceramicine F, and the differences between them were reminiscent of the differences between ceramicine B and ceramicine N with a 14,15-epoxy moiety in ceramicine B [13]. Thus, **1** was suggested to be the 14,15-epoxy derivative of ceramicine F. This suggestion was verified by analyzing the 2D NMR data (Fig. 2); in particular, the HMBC correlations of H_3_-30 and H_3_-18 to C-14, H_3_-18 to C-12, C-13, and C-17, and H_2_-16 to C-13 and C-14, verified the presence of an epoxide ring at C-14 and C-15. Thus, **1** was deduced to be a new limonoid with a cyclopentanone[α]phenanthrene ring system and a furan ring at C-17 as shown in Fig. 2.

**Table 1:** ^1^H and ^13^C NMR data of **1**–**6** in CDCl_3_

No	**1**		**2**		**3**	
	δ_H_ (*J*_,_ Hz)	δ_C_	δ_H_ (*J*_,_ Hz)	δ_C_	δ_H_ (*J*_,_ Hz)	δ_C_
1		202.4		203.4		202.3
2	5.95 (1H, d, 10.0)	128.9	5.83 (1H, d, 10.2)	126.9	5.92 (1H, d, 10.2)	129.5
3	6.09 (1H, d, 10.0)	144.4	6.33 (1H, d, 10.2)	146.8	6.22 (1H, d, 10.2)	149.7
4		52.1		48.0		57.9
5	2.99 (1H, d, 10.6)	43.6	3.51 (1H, d, 11.5)	42.8	3.21 (1H, d, 11.5)	42.3
6	3.89 (1H, dd, 10.6, 10.6)	66.3	3.86 (1H, dd, 11.5, 1.7)	66.7	5.42 (1H, brd, 11.5)	66.9
7	3.50 (1H, m)	73.6	3.47 (1H, brs)	74.2	3.83 (1H, brs)	74.1
8		40.6		40.3		44.2
9	2.74 (1H, dd, 9.3, 8.8)	33.5	2.76 (1H, m)	34.2	2.71 (1H, m)	32.0
10		48.2		48.9		50.6
11a	1.67 (1H, m)	18.4	1.31 (1H, m)	16.9	1.60 (1H, m)	17.8
11b	2.63 (1H, m)		1.60 (1H, m)		2.50 (1H, m)	
12a	1.72 (1H, m)	33.5	1.70 (1H, m)	33.5	1.60 (1H, m)	33.0
12b	1.85 (1H, m)		1.86 (1H, m)		1.93 (1H, m)	
13		43.9		43.8		47.0
14		81.4		81.4		159.5
15	3.72 (1H, s)	63.8	3.71 (1H, m)	63.5	5.53 (1H, s)	120.4
16a	1.90 (1H, m)	33.2	1.87 (1H, m)	33.3	2.39 (1H, m)	34.3
16b	2.26 (1H, m)		2.25 (1H, m)		2.51 (1H, m)	
17	3.12 (1H, t, 9.3)	52.1	3.11 (1H, dd, 9.7, 8.8)	52.5	2.85 (1H, dd, 10.6, 8.1)	52.0
18	0.84 (3H, s)	18.7	0.84 (3H, s)	18.8	0.90 (3H, s)	21.7
19	1.34 (3H, s)	16.9	1.31 (3H, s)	16.7	1.35 (3H, s)	14.7
20		124.8		126.9		124.4
21	7.16 (1H, s)	139.5	7.16 (1H, s)	139.5	7.25 (1H, s)	139.7
22	6.24 (1H, s)	110.9	6.24 (1H, s)	110.8	6.29 (1H, s)	111.0
23	7.34 (1H, s)	142.8	7.33 (1H, s)	142.4	7.37 (1H, s)	142.6
28	9.28 (1H, s)	200.7		175.7		
29a	1.54 (3H, s)	15.7	1.61 (3H, s)	17.1	2.81 (1H, s)	50.0
29b					3.37 (1H, s)	
30	1.21 (3H, s)	22.8	1.19 (3H, s)	23.0	1.23 (3H, s)	25.7
1′			3.70 (3H, s)	52.9	2.12 (3H, s)	21.4
2′						170.8
3′						

No	**4**		**5**		**6**	
	δ_H_ (*J*_,_ Hz)	δ_C_	δ_H_ (*J*_,_ Hz)	δ_C_	δ_H_ (*J*_,_ Hz)	δ_C_
1		202.9		202.2		202.8
2	5.81 (1H, d, 9.8)	130.4	6.01 (1H, d, 10.0)	129.6	5.86 (1H, d, 10.1)	126.6
3	6.92 (1H, d, 9.8)	150.6	5.98 (1H, d, 10.0)	143.2	6.25 (1H, d, 10.1)	146.0
4		46.0		51.9		48.9
5	3.06 (1H, d, 12.8)	44.3	3.23 (1H, d, 12.2)	40.2	3.65 (1H, d, 12.3)	41.2
6	4.38 (1H, dd, 12.8, 2.7)	75.1	5.33 (1H, dd, 12.2, 2.3)	69.6	5.36 (1H, dd, 12.3, 2.2)	69.8
7	4.22 (1H, d, 2.7)	71.7	4.10 (1H, d, 2.3)	72.6	4.05 (1H, brs)	72.9
8		46.8		44.6		44.7
9	2.45 (1H, m)	36.1	2.64 (1H, m)	33.2	2.62 (1H, m)	33.6
10		47.3		47.8		47.5
11a	1.80 (1H, m)	17.7	1.63 (1H, m)	18.1	1.60 (1H, m)	18.1
11b	2.50 (1H, m)		2.50 (1H, m)		2.54 (1H, m)	
12a	1.60 (1H, m)	33.1	1.65 (1H, m)	32.9	1.61 (1H, m)	33.0
12b	1.89 (1H, m)		1.95 (1H, m)		1.93 (1H, m)	
13		47.0		47.1		47.2
14		159.7		159.8		160.3
15	5.59 (1H, s)	120.1	5.57 (1H, s)	120.8	5.54 (1H, s)	120.6
16a	2.39 (1H, m)	34.3	2.42 (1H, m)	34.3	2.40 (1H, m)	34.3
16b	2.54 (1H, m)		2.52 (1H, m)		2.50 (1H, m)	
17	2.84 (1H, dd, 10.8, 7.4)	51.9	2.88 (1H, dd, 10.6, 7.7)	52.2	2.86 (1H, dd, 10.8, 7.8)	51.9
18	0.82 (3H, s)	21.4	0.92 (3H, s)	21.7	0.90 (3H, s)	21.7
19	1.17 (3H, s)	14.9	1.36 (3H, s)	16.7	1.33 (3H, s)	16.4
20		124.6		124.4		124.2
21	7.24 (1H, s)	139.6	7.26 (1H, s)	139.7	7.26 (1H, s)	139.7
22	6.29 (1H, s)	111.1	6.30 (1H, s)	111.0	6.30 (1H, s)	111.0
23	7.37 (1H, s)	142.5	7.39 (1H, s)	142.7	7.38 (1H, s)	142.7
28	4.69 (1H, s)	111.4	9.18 (1H, s)	199.7		175.3
29	1.26 (3H, s)	18.4	1.30 (1H, s)	14.6	1.39 (1H, s)	16.6
30	1.14 (3H, s)	26.1	1.28 (3H, s)	25.9	1.25 (3H, s)	26.1
1′	3.40 (3H, s)	55.2			3.72 (3H, s)	52.8
2′			1.99 (3H, s)	20.8	2.04 (3H, s)	21.0
3′				170.2		171.1

The relative configuration of **1** was assigned through analyses of the NOESY data (Fig. 2) and ^1^H–^1^H coupling constant data. The NOESY correlations of H-5/H-9 and H-28 suggested the *α*-orientation of H-5, H-9, and CHO-28. The value of ^3^*J*_H5/H6_ = 10.6 Hz and ^3^*J*_H6/H7_ = 0 Hz suggested the orientation of both H-6 and H-7 to be β. The ROESY correlations of H-6/H_3_-19, H_3_-29, and H_3_-30 and H-9/H_3_-18 further confirmed the *α*-orientation of CH_3_-18, and also indicated that CH_3_-19, CH_3_-29, and CH_3_-30 were *β*-oriented. On the other hand, the relative configurations of C-13 and C-17 were deduced from the NOESY correlations of H-17/H-12a (Fig. 2). The stereochemistry of the 14,15-*α*-epoxy ring was elucidated to be as shown in Fig. 2 by the presence of NOESY correlations of H_3_-30 and H-15.

Ceramicine V (**2**) was obtained as white amorphous solid, and the HRESIMS displayed a pseudomolecular ion peak at 493.2204 (M + Na)^+^ corresponding to the molecular formula C_27_H_34_O_7_. IR absorptions indicated the presence of hydroxy (3443 cm^−1^) and carbonyl (1732 and 1684 cm^−1^) groups. Analyses of the ^1^H and ^13^C NMR data (Table 1) revealed the structural similarity of **1** and **2**. The differences observed in their NMR data were the appearance of a methoxy carbonyl signals (C-28: *δ*_C_ 175.7, CH_3_-1’: *δ*_C_ 52.9, *δ*_H_ 3.70) in **2** instead of an aldehyde signal (C-28: *δ*_C_ 200.7, *δ*_H_ 9.28) detected in **1**. Thus, **2** was deduced to be a methoxy carbonyl derivative of **1**. Analysis of the 2D NMR (^1^H–^1^H COSY, HSQC, HMBC, and NOESY) data further supported this deduction (see Supplementary Information).

Ceramicine W (**3**) was obtained as white amorphous powder, and the HRESIMS displayed a pseudomolecular ion peak at 475.2083 (M + Na)^+^ corresponding to the molecular formula C_27_H_32_O_6._ IR absorptions indicated the presence of hydroxy (3444 cm^−1^) and carbonyl (1728 and 1685 cm^−1^) groups. Analyses of the ^1^H and ^13^C NMR data (Table 1) revealed the structural similarity of **3** and ceramicine Q [11], and **3** was assumed to be a 6-*O*-acetyl derivative of ceramicine Q, since one additional acetyl methyl signal was observed [C-2′: *δ*_C_ 170.8, CH_3_-1′: *δ*_C_ 21.4, *δ*_H_ 2.12] in **3**]. Analyses of the 2D NMR (^1^H-^1^H COSY, HSQC, HMBC, and NOESY) data further support this assumption (see Supplementary Information).

Ceramicine X (**4**) was revealed to have the molecular formula C_27_H_34_O_5_ by HRESIMS. Its ^1^H and ^13^C NMR data are highly similar to ceramicine B [19]. However, the NMR data of **4** suggested that the 2-methoxytetrahydrofuran moiety [*δ*_H_ 4.69 (s), *δ*_C_ 111.4; *δ*_H_ 3.40 (3H, s), *δ*_C_ 55.2] at C-28 was observed instead of the oxymethylene function in ceramicine B. The HMBC correlations of H_3_-1′ and H_3_-29 to C-28 (*δ*_C_ 111.4) in SI supported its functionality in the structure of **4**. Finally, the NOESY correlation between H-28 and H_3_-29 shown in SI confirmed the α orientation of the methoxy group at C-28.

By HRESITOFMS, ceramicines Y (**5**) and Z (**6**) were revealed to have the molecular formula C_28_H_34_O_6_ and C_29_H_36_O_7_, respectively. Their NMR data are also highly similar to **3**, differing only on the signals assigned to the presence of one aldehyde group in **5** [*δ*_H_ 9.18 (s), *δ*_C_ 199.7] and one methoxy carbonyl group in **6** [*δ*_H_ 3.72 (s), *δ*_C_ 52.8; *δ*_C_ 175.3] in addition of a methyl group [*δ*_H_ 1.30 (s), *δ*_C_ 14.6 in **5**; *δ*_H_ 1.39 (s), *δ*_C_ 16.6 in **6**], in place of the oxy-methylene protons in **3** (the presence of one epoxide group) [*δ*_H_ 2.81 (s) and 3.37 (s), *δ*_C_ 50.0]. IR absorptions implied the presence of α,β-unsaturated ketone (1688 cm^−1^), hydroxy (3452 cm^−1^ in **5** and 3515 cm^−1^ in **6**), and aldehyde, acetyl (1729 cm^−1^) and/or methoxy carbonyl (1734 cm^−1^) groups. Analysis of the NMR data revealed that the presence of a cyclopentanone[α]phenanthren ring system with a β-furyl ring at C-17.

The planar structures of **5** and **6** were deduced from the ^1^H-^1^H COSY correlations and the especially HMBC correlations of H_3_-29 to C-3, C-4, C-5 and C-28 as shown in SI. The relative configurations of **5** and **6** were then deduced from the NOESY correlations as shown in SI. Thus, the structures of **5** and **6** were proposed to be as shown in Fig. 1.

Considering that **1**–**6** were isolated from the same extract as ceramicine B [19], their absolute configurations were assumed to be similar to ceramicine B based on the biogenetic relationships. In addition, CD spectra similarities of **1** and** 5** to ceramicine F; **2** and **6** to ceramicine G; **3** to ceramicine Q; and **4** to ceramicine B further supported the assumption.

## Antimalarial activity

We have reported some the antimalarial activity of several ceramicines against *P. falciparum* 3D7 in vitro [11, 19, 20]. Thus, we tested the antimalarial activity of ceramicines U–Z (**1**–**6**) against *Plasmodium falciparum* 3D7 strain (Table 2). The assay showed that ceramicine W (**3**) had potent in vitro antimalarial activity [the half-maximal (50%) inhibitory concentration (IC_50_) = 1.2 µM, whereas the others did not (> 5.0 µM)].

**Table 2:** Antimalarial activity of **1**–**37** against *Plasmodium falciparum* 3D7 strain

	IC_50_ (µM)	IC_50_ (µM)	
Ceramicine U (**1**)	> 5.0	Ceramicine M (**19**)	0.8
Ceramicine V (**2**)	> 5.0	Ceramicine N (**20**)	2.1
Ceramicine W (**3**)	1.2	Ceramicine O (**21**)	1.3
Ceramicine X (**4**)	> 5.0	Ceramicine P (**22**)	2.5
Ceramicine Y (**5**)	> 5.0	Ceramicine Q (**23**)	> 5.0
Ceramicine Z (**6**)	> 5.0	Ceramicine R (**24**)	2.8
Ceramicine A (**7**)	4.5	Ceramicine S (**25**)	> 5.0
Ceramicine B (**8**)	> 5.0	Ceramicine T (**26**)	> 5.0
Ceramicine C (**9**)	2.7	**27**	2.6
Ceramicine D (**10**)	2.7	**28**	> 5.0
Ceramicine E (**11**)	> 5.0	**29**	> 5.0
Ceramicine F (**12**)	> 5.0	**30**	0.89
Ceramicine G (**13**)	> 5.0	**31**	0.70
ceramicine H (**14**)	1.0	**32**	> 5.0
Ceramicine I (**15**)	2.1	**33**	> 5.0
Ceramicine J (**16**)	> 5.0	**34**	0.82
Ceramicine K (**17**)	–	**35**	4.5
Ceramicine L (**18**)	> 5.0	**36**	0.81
		**37**	> 5.0

**Fig. 3:**
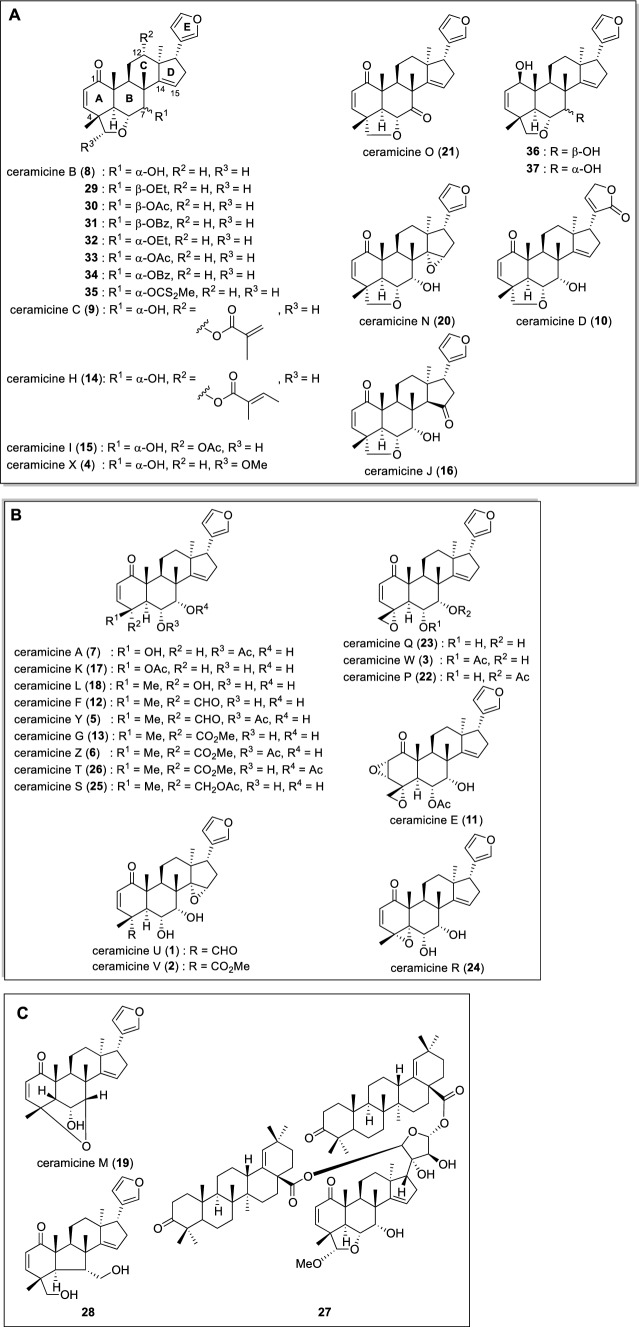
Structures of ceramicines and their derivatives

**Fig. 4:**
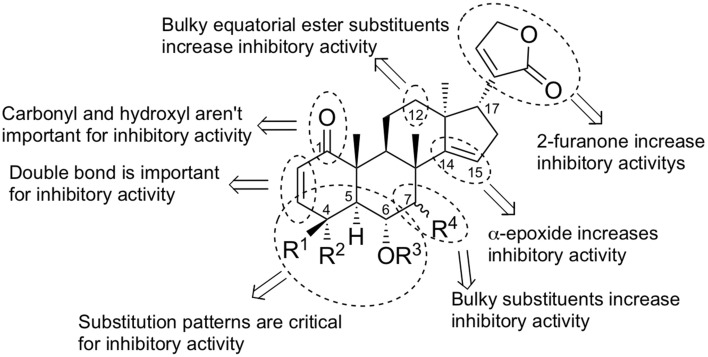
SAR of ceramicines for antimalarial activity

In addition to the data of the newly isolated compounds, the antimalarial activities of other ceramicines and synthesized ceramicine B derivatives [16] are also shown in Table 2. Considering the number of the data we have on hand, we then examined the structure–antimalarial activity relationships (SAR) of the ceramicines. For easier comparison, the compounds (**1**–**37**) were grouped according to their structures (Fig. 3). The structure–activity relationship of the ceramicines is summarized in Fig. 4.

First, we examined the effect of the substituents at C-7. Based on the antimalarial activity of **36** and **37**; **30** and **33**; **31** and **34**; and **21** and **8**, it can be inferred that substituents at C-7 with an equatorial orientation are preferred more than those with an axial orientation. In addition, based on the activity of compounds **8**, **22**, **23,** and **29**–**35**, it is clear that a bulkier substituent at C-7, regardless of the orientation, may enhance the antimalarial activity of the ceramicines.

Next, the effect of the substituents at C-4–C-6 was examined. Based on the activity of **3**, **5–7**, **12–13**, **18**, **22–26**, and **33**, it is clear that the substituents at C-4–C-6 play some role in the antimalarial activity of the ceramicines. Notably, the activities of **3** and **23** indicate that the α-equatorially oriented ester group at C-6 is preferred over the hydroxy group; substituent at C-6 may have a similar effect to those at C-7, i.e., bulky > small and equatorial > axial; unfortunately, the current set of data is not enough to confirm this hypothesis. Finally, the activity of **22**, **26** and **33**;** 3** and **5–7**; **24**, **12**, **13**, **18**, **23** and **25**, seems to indicate that a less bulky substituent or an α-oriented epoxide group at C-4 is preferred.

Next, we examined **8**, **9**, **14**, and **15** which differ only in substituents at C-12. **8** showed no antimalarial activity, while **9**, **14**, and **15** showed antimalarial activity, with **14** showing the highest activity. From this result, we inferred that an α-equatorially oriented bulky ester group at C-12 may enhance the antimalarial activity.

Finally, we examined the effect of the α,β-unsaturated ketone, C-14 double bonds, and the furan groups. Regarding the α,β-unsaturated ketone group, only the double bond contributes to the antimalarial activity as can be inferred from the activity of **3** and **11**; **21** and **36**. Oxidation of the double bond at C-14 to an epoxide (**8** to **20**) led to increased activity, but further oxidation to a ketone (**16**) results in decreased activity. Regarding the furan unit, it is interesting to note that unlike the pattern we observed for the anti-lipid droplet accumulation inhibitory activity and anti-melanin deposition activity of the ceramicines [14, 16], the oxidation of the furan unit to a furanone (**8** to **10**) led to increased activity. We believe that the activity of the ceramicines can be further optimized by modifying ring D and E, and further study is needed to clarify substituent effect on both rings.

The structures of **19**, **27,** and **28** are significantly different from the rest of the ceramicines, and thus, it is difficult to draw any conclusion regarding the structure–activity relationships. However, based on the above discussion and the activity of **19** and **27**, it appears that ring B and the functional groups in the vicinity of rings B and C are critical for the antimalarial activity of the ceramicines.

## Experimental section

***General experimental procedures*** Optical rotations were measured on a JASCO DIP-1000 polarimeter. UV spectra were recorded on a Shimadzu UVmini-1240 spectrophotometer and IR spectra on a JASCO FT/IR-4100 spectrophotometer. High-resolution ESI-MS were obtained on a JMS-T100LP (JEOL). 1D and 2D NMR spectra were measured on a 400 MHz (Brucker AV-400) or 600 MHz (JEOL ECZ600) spectrometer at 300 K, while ^13^C NMR spectra were on a 100 MHz or 150 MHz spectrometer. The residual solvent peaks were used as internal standards (δ_H_ 7.26 and δ_C_ 77.0 for CDCl_3_).

***Material*** The barks of *C*. *ceramicus* were collected in Terengganu, Malaysia in July 2013. The botanical identification was made by Prof. A. Hamid A. Hadi, University of Malaya. Voucher specimens (No. HOSHI13CCB) are deposited in the Department of Pharmacognosy Hoshi University.

***Extraction and isolation*** The barks of *C*. *ceramicus* (8 kg) were extracted with MeOH to obtain 1.43 kg of extract. The MeOH extract was successively partitioned with *n*-hexane, EtOAc, *n*-BuOH, and water. The *n*-hexane-soluble materials were separated further by silica gel column chromatography (*n*-hexane/EtOAc 1:0→1:1, CHCl_3_/MeOH 1:0→0:1) to obtain 10 fractions (A–J).

Fraction G was separated further with LH-20 gel column (CHCl_3_/MeOH 1:1) to obtain 5 fractions (G-1–G-5). Fraction G-4 was then separated by HPLC (Shiseido ODS MGII 20 × 250 mm, 77.5% aqueous MeOH at 9.5 mL/min, UV detection at 210 nm) into 11 fractions (G-4-a–G-4-k). Fraction G-4-c was pure ceramicine W (**3**: 7.1 mg, 8.9 × 10^–5^%, *t*_R_ 19.1 min). Separation of fraction G-4-g by HPLC (Shiseido ODS MGII 20 × 250 mm, 70% aqueous MeOH at 9.5 mL/min, UV detection at 210 nm) yielded ceramicine X (**4**: 10.8 mg, 13.5 × 10^–5^%, *t*_R_ 31.9 min) and a mixture of ceramicine Y (**5**) and ceramicine Z (**6**). The mixture was subjected to passage over silica gel column chromatography (*n*-hexane/EtOAc 7:3→1:1) to obtain pure ceramicine Y (**5**: 1.6 mg, 2.0 × 10^–5^%) and ceramicine Z (**6**: 1.9 mg, 2.4 × 10^–5^%).

Fraction I was separated further with an ODS silica gel column (MeOH/H_2_O 7:3→1:0, acetone) to obtain 6 fractions (I-1–I-6). Fraction I-2 was then separated by HPLC (Shiseido ODS MGII 30 × 250 mm, 75% aqueous MeOH at 8.0 mL/min, UV detection at 210 nm) into 9 fractions (I-2-a–I-2-i). Separation of fraction I-2-d by HPLC (COSMOSIL Cholester 10 × 250 mm, 50% aqueous MeCN at 2 mL/min, UV detection at 210 nm) yielded ceramicine U (**1**: 2.0 mg, 2.5 × 10^–5^%, *t*_R_ 44 min) and ceramicine V (**2**: 1.5 mg, 1.9 × 10^–5^%, 51 min).

**Ceramicine U (1)**: white amorphous solid; [α]_D_^31^ -63 (*c* 1.0, CHCl_3_); UV (MeOH) *λ*_max_ (*ε*) 204 (8140) nm; CD (MeOH) *λ*_max_ (Δ*ε*) 301 (-5.21) and 231 (+ 7.82) nm; IR (Zn-Se) *ν*_max_ 3371, 1721 and 1685 cm^–1^; ^1^H and ^13^C NMR data (Table 1); ESI-MS *m/z* 463 (M+Na)^+^; HRESIMS *m/z* 463.2127 (M+Na)^+^; calcd for C_26_H_32_O_6_Na, 463.2097).

**Ceramicine V (2)**: white amorphous solid; [α]_D_^31^ + 6.6 (*c* 1.0, CHCl_3_); UV (MeOH) *λ*_max_ (*ε*) 204 (2450) nm; CD (MeOH) *λ*_max_ (Δ*ε*) 336 (− 0.47), 266 (0.17), 234 (− 2.72) and 207 (3.82) nm; IR (Zn-Se) *ν*_max_ 3443, 1732 and 1684 cm^–1^; ^1^H and ^13^C NMR data (Table 1); ESI-MS *m/z* 493 (M+Na)^+^; HRESIMS *m/z* 493.2204 (M+Na)^+^; calcd for C_27_H_34_O_7_Na, 493.2202).

**Ceramicine W (3)**: white amorphous solid; [α]_D_^30^ + 58 (*c* 1.0, CHCl_3_); UV (MeOH) *λ*_max_ (*ε*) 205 (9460) nm; CD (MeOH) *λ*_max_ (Δ*ε*) 340 (− 1.52), 246 (− 0.54) and 217 (8.69) nm; IR (Zn-Se) *ν*_max_ 3444, 1728 and 1685 cm^–1^; ^1^H and ^13^C NMR data (Table 1); ESI-MS *m/z* 475 (M+Na)^+^; HRESIMS *m/z* 475.2083 (M+Na)^+^; calcd for C_27_H_32_O_6_Na, 475.2097).

**Ceramicine X (4)**: white amorphous solid; [α]_D_^29^ + 89 (*c* 1.0, CHCl_3_); UV (MeOH) *λ*_max_ (*ε*) 206 (12,600) nm; CD (MeOH) *λ*_max_ (Δ*ε*) 332 (− 0.45) and 218 (7.51) nm; IR (Zn-Se) *ν*_max_ 3432 and 1672 cm^–1^; ^1^H and ^13^C NMR data (Table 1); ESI-MS *m/z* 461 (M+Na)^+^; HRESIMS *m/z* 461.2325 (M+Na)^+^; calcd for C_27_H_34_O_5_Na, 461.2304).

**Ceramicine Y (5)**: White amorphous solid; [α]_D_^25^ -29° (*c* 0.25, CHCl_3_); IR (Zn-Se) *ν*_max_ 3452, 1729 and 1688 cm^−1^; UV (MeOH) *λ*_max_ (*ε*) 203.5 (5527) nm; CD (MeOH) *λ*_max_ (Δ*ε*) 232 (24.51) and 301 (− 15.91) nm; ^1^H and ^13^C NMR, see Table 1; ESI-MS *m/z* 489 (M + Na)^+^; HRESIMS *m/z* 489.2250 (M+Na)^+^; calcd. for C_28_H_34_O_6_Na, 489.2253.

**Ceramicine Z (6)**: White amorphous solid; [α]_D_^26^-12° (*c* 0.25, CHCl_3_); IR (Zn-Se) *ν*_max_ 3515, 1734 and 1688 cm^−1^; UV (MeOH) *λ*_max_ (*ε*) 203.5 (4246) nm; CD (MeOH) *λ*_max_ (Δ*ε*) 208 (38.66), 233 (− 38.09) and 335 (− 5.03) nm; ^1^H and ^13^C NMR, see Table 1; ESI-MS *m/z* 519 (M+Na)^+^; HRESIMS *m/z* 519.2370 (M+Na)^+^; calcd. for C_29_H_36_O_7_Na, 519.2359.

***Parasite strain culture*** *P. falciparum* laboratory strain 3D7 was obtained from Prof. Masatsugu Kimura (Osaka City University, Osaka, Japan). For the assessment of antimalarial activity of the compounds in vitro, the parasites were cultured in Roswell Park Memorial Institute (RPMI) 1640 medium supplemented with 0.5 g/L l-glutamine, 5.96 g/L HEPES, 2 g/L sodium bicarbonate (NaHCO_3_), 50 mg/L hypoxanthine, 10 mg/L gentamicin, 10% heat-inactivated human serum, and red blood cells (RBCs) at a 3% hematocrit in an atmosphere of 5% CO_2_, 5% O_2_, and 90% N_2_ at 37 ℃ as previously described [26]. Ring-form parasites were collected using the sorbitol synchronization technique [27]. Briefly, the cultured parasites were collected by centrifugation at 840 g for 5 min at room temperature and suspended in a fivefold volume of 5% D-sorbitol (Nacalai Tesque, Kyoto, Japan) for 10 min at room temperature, and then, they were washed twice with RPMI 1640 medium to remove the D-sorbitol. The utilization of blood samples of healthy Japanese volunteers for the parasite culture was approved by the institutional review committee of the Research Institute for Microbial Diseases (RIMD), Osaka University (Approval Number: 22–3).

***Antimalarial activity*** Ring-form-synchronized parasites were cultured with compounds **1**–**37** at sequentially decreasing concentrations (5, 1.5, 0.5, and 0.15 µM) for 48 h for the flow cytometric analysis using an automated hematology analyzer, XN-30. The XN-30 analyzer was equipped with a prototype algorithm for cultured falciparum parasites (prototype; software version: 01–03, (build 16)) and used specific reagents (CELLPACK DCL, SULFOLYSER, Lysercell M, and Fluorocell M) (Sysmex, Kobe, Japan) [28–30]. Approximately 100 µL of the culture suspension diluted with 100 µL phosphate-buffered saline was added to a BD Microtainer MAP Microtube for Automated Process K_2_ EDTA 1.0 mg tube (Becton Dickinson and Co., Franklin Lakes, NJ, USA) and loaded onto the XN-30 analyzer with an auto-sampler as described in the instrument manual (Sysmex). The parasitemia (MI-RBC%) was automatically reported. Then, 0.5% DMSO alone or containing 5 µM artemisinin used as the negative and positive controls, respectively. The growth inhibition (GI) rate was calculated from the MI-RBC% according to the following equation:

$$ {\text{GI }}\left( \% \right)\, = \,{1}00{-\!\!-}{{\left( {{\text{test sample}}{-\!\!-}{\text{positive control}}} \right)} \mathord{\left/ {\vphantom {{\left( {{\text{test sample}}{-\!\!-}{\text{positive control}}} \right)} {\left( {{\text{negative control}}{-\!\!-}{\text{positive control}}} \right)}}} \right. \kern-0pt} {\left( {{\text{negative control}}{-\!\!-}{\text{positive control}}} \right)}} \times \,{1}00. $$

The IC_50_ was calculated from GI (%) using GraphPad Prism version 5.0 (GraphPad Prism Software, San Diego, CA, USA) [27].

## Supplementary Information

Below is the link to the electronic supplementary material.

Supplementary file 1 (PDF 5699 KB)
