# Correction: Ceramicines U–Z from *Chisocheton ceramicus* and structure–antimalarial activity relationship study

**DOI:** 10.1007/s11418-025-01898-3

**Published:** 2025-04-03

**Authors:** Alfarius Eko Nugroho, Tomoyuki Komuro, Takuya Kawaguchi, Yusuke Shindo, Chin Piow Wong, Yusuke Hirasawa, Toshio Kaneda, Takahiro Tougan, Toshihiro Horii, A. Hamid A. Hadi, Hiroshi Morita

**Affiliations:** 1 Faculty of Pharmaceutical Sciences, Hoshi University, Ebara 2-4-41 Shinagawa-Ku, Tokyo, 142-8501 Japan; 2 Research Center for Infectious Disease Control, Research Institute for Microbial Diseases, Osaka University, 3-1 Yamadaoka, Suita, Osaka 565-0871 Japan; 3 Department of Malaria Vaccine Development, Research Institute for Microbial Diseases, Osaka University, 3-1 Yamadaoka, Suita, Osaka 565-0871 Japan; 4 Department of Chemistry, Faculty of Science, University of Malaya, 50603 Kuala Lumpur, Malaysia

**Correction: Journal of Natural Medicines (2024) 78:68–77** 10.1007/s11418-023-01746-2

The article “Ceramicines U–Z from *Chisocheton ceramicus* and structure–antimalarial activity relationship study”, written by Alfarius Eko Nugroho, Tomoyuki Komuro, Takuya Kawaguchi, Yusuke Shindo, Chin Piow Wong, Yusuke Hirasawa, Toshio Kaneda, Takahiro Tougan, Toshihiro Horii, A. Hamid A. Hadi and Hiroshi Morita, was originally published under exclusive license to The Author(s) under exclusive licence to The Japanese Society of Pharmacognosy. As a result of the subsequent decision to publish the article under the open access model, the article’s copyright notice was changed on 5 March 2025 to © The Author(s) 2025 and the article is now distributed under a Creative Commons Attribution 4.0 International License, which permits use, sharing, adaptation, distribution and reproduction in any medium or format, as long as you give appropriate credit to the original author(s) and the source, provide a link to the Creative Commons licence, and indicate if changes were made. The images or other third party material in this article are included in the article’s Creative Commons licence, unless indicated otherwise in a credit line to the material. If material is not included in the article’s Creative Commons licence and your intended use is not permitted by statutory regulation or exceeds the permitted use, you will need to obtain permission directly from the copyright holder. To view a copy of this licence, visit http://creativecommons.org/licenses/by/4.0.

The original article has been corrected.

