# Supplementary material for: Ceramicines U–Z from Chisocheton ceramicus and structure–antimalarial activity relationship study
Source: J Nat Med. 2023 Sep 10;78(1):68–77. doi: 10.1007/s11418-023-01746-2 (PMC11968507; doi:10.1007/s11418-023-01746-2)
Supplement: Supplementary file 1 — (PDF 5699 KB) [file 11418_2023_1746_MOESM1_ESM.pdf]

Ceramicines U-Z from *Chisocheton ceramicus* and Structure – Antiplasmodial Activity Relationship Study

Alfarius Eko Nugroho[1], Tomoyuki Komuro[1], Takuya Kawaguchi[1], Yusuke Shindo[1], Chin Piow Wong[1], Yusuke Hirasawa[1,*], Toshio Kaneda[1], Takahiro Tougan[2c], Toshihiro Horii[3d], A. Hamid A., Hadi[4], and Hiroshi Morita[1,*]

[1]*Faculty of Pharmaceutical Sciences, Hoshi University, Ebara 2-4-41 Shinagawa-ku, Tokyo 142-8501, Japan;*

[2c]*Research Center for Infectious Disease Control, Research Institute for Microbial Diseases, Osaka University, 3-1 Yamadaoka, Suita, Osaka 565-0871, Japan*

[3d]*Department of Malaria Vaccine DevelopmentDepartment of Molecular Protozoology, Research Institute for Microbial Diseases, Osaka University, 3-1 Yamadaoka, Suita, Osaka 565-0871, Japan*

[4]*Department of Chemistry, Faculty of Science, University of Malaya, 50603 Kuala Lumpur, Malaysia*

Supporting Information

Figure S1.	Selected 2D NMR correlations of **2**.	S3
Figure S2.	Selected 2D NMR correlations of **3**.	S4
Figure S3.	Selected 2D NMR correlations of **4**.	S5
Figure S4.	Selected 2D NMR correlations of **5**.	S6
Figure S5.	Selected 2D NMR correlations of **6**.	S7
Figure S6.	^{1}H NMR spectrum of ceramicine U (**1**) in $CDCl_3$.	S8
Figure S7.	^{13}C NMR spectrum of ceramicine U (**1**) in $CDCl_3$.	S9
Figure S8.	1H-1H COSY spectrum of ceramicine U (**1**) in $CDCl_3$.	S10
Figure S9.	HSQC spectrum of ceramicine U (**1**) in $CDCl_3$.	S11
Figure S10.	HMBC spectrum of ceramicine U (**1**) in $CDCl_3$.	S12
Figure S11.	NOESY spectrum of ceramicine U (**1**) in $CDCl_3$.	S13
Figure S12.	^{1}H NMR spectrum of ceramicine V (**2**) in $CDCl_3$.	S14
Figure S13.	^{13}C NMR spectrum of ceramicine V (**2**) in $CDCl_3$.	S15
Figure S14.	^{1}H-^{1}H COSY spectrum of ceramicine V (**2**) in $CDCl_3$.	S16
Figure S15.	HSQC spectrum of ceramicine V (**2**) in $CDCl_3$.	S17
Figure S16.	HMBC spectrum of ceramicine V (**2**) in $CDCl_3$.	S18
Figure S17.	NOESY spectrum of ceramicine V (**2**) in $CDCl_3$.	S19
Figure S18.	^{1}H NMR spectrum of ceramicine W (**3**) in $CDCl_3$.	S20
Figure S19.	^{13}C NMR spectrum of ceramicine W (**3**) in $CDCl_3$.	S21
Figure S20.	^{1}H-^{1}H COSY spectrum of ceramicine W (**3**) in $CDCl_3$.	S22
Figure S21.	HSQC spectrum of ceramicine W (**3**) in $CDCl_3$.	S23

Figure S22.	HMBC spectrum of ceramicine W (**3**) in $CDCl_3$.	S24
Figure S23.	NOESY spectrum of ceramicine W (**3**) in $CDCl_3$.	S25
Figure S24.	^{1}H NMR spectrum of ceramicine X (**4**) in $CDCl_3$.	S26
Figure S25.	^{13}C NMR spectrum of ceramicine X (**4**) in $CDCl_3$.	S27
Figure S26.	^{1}H-^{1}H COSY spectrum of ceramicine X (**4**) in $CDCl_3$.	S28
Figure S27.	HSQC spectrum of ceramicine X (**4**) in $CDCl_3$.	S29
Figure S28.	HMBC spectrum of ceramicine X (**4**) in $CDCl_3$.	S30
Figure S29.	NOESY spectrum of ceramicine X (**4**) in $CDCl_3$.	S31
Figure S30.	^{1}H NMR spectrum of ceramicine Y (**5**) in $CDCl_3$.	S32
Figure S31.	^{13}C NMR spectrum of ceramicine Y (**5**) in $CDCl_3$.	S33
Figure S32.	^{1}H-^{1}H COSY spectrum of ceramicine Y (**5**) in $CDCl_3$.	S34
Figure S33.	HSQC spectrum of ceramicine Y (**5**) in $CDCl_3$.	S35
Figure S34.	HMBC spectrum of ceramicine Y (**5**) in $CDCl_3$.	S36
Figure S35.	NOESY spectrum of ceramicine Y (**5**) in $CDCl_3$.	S37
Figure S36.	^{1}H NMR spectrum of ceramicine Z (**6**) in $CDCl_3$.	S38
Figure S37.	^{13}C NMR spectrum of ceramicine Z (**6**) in $CDCl_3$.	S39
Figure S38.	^{1}H-^{1}H COSY spectrum of ceramicine Z (**6**) in $CDCl_3$.	S40
Figure S39.	HSQC spectrum of ceramicine Z (**6**) in $CDCl_3$.	S41
Figure S40.	HMBC spectrum of ceramicine Z (**6**) in $CDCl_3$.	S42
Figure S41.	NOESY spectrum of ceramicine Z (**6**) in $CDCl_3$.	S43

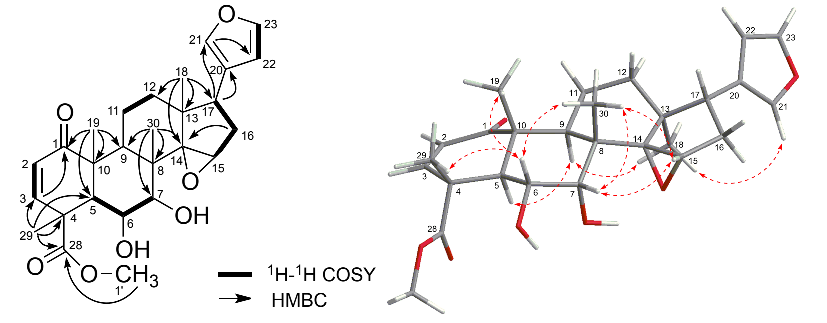

Figure S1. Selected 2D NMR correlations of **2**.

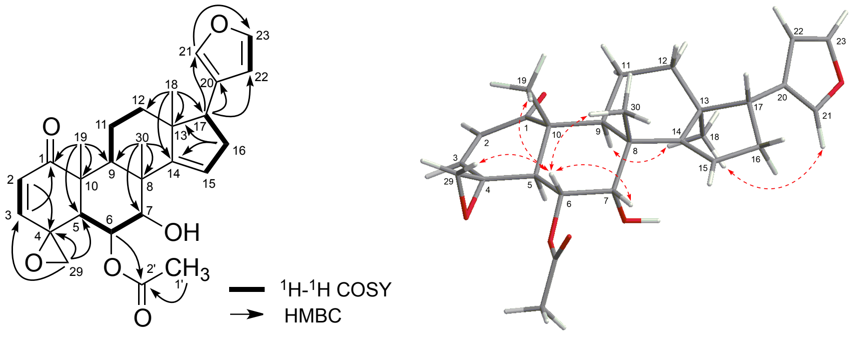

Figure S2. Selected 2D NMR correlations of **3**.

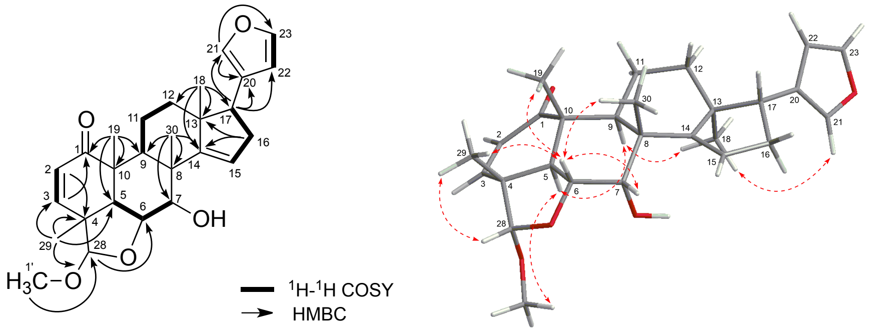

Figure S3. Selected 2D NMR correlations of **4**.

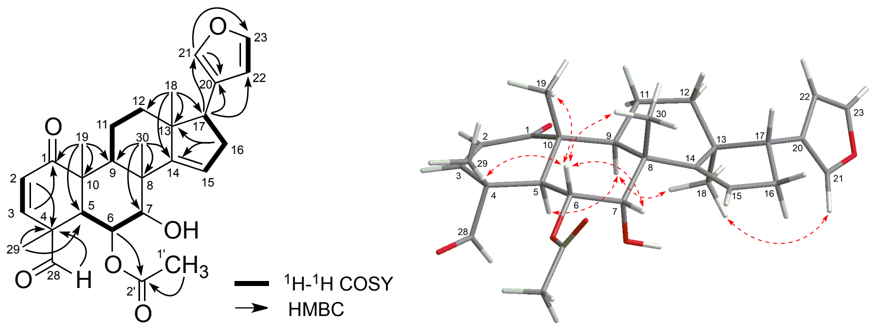

Figure S4. Selected 2D NMR correlations of **5**.

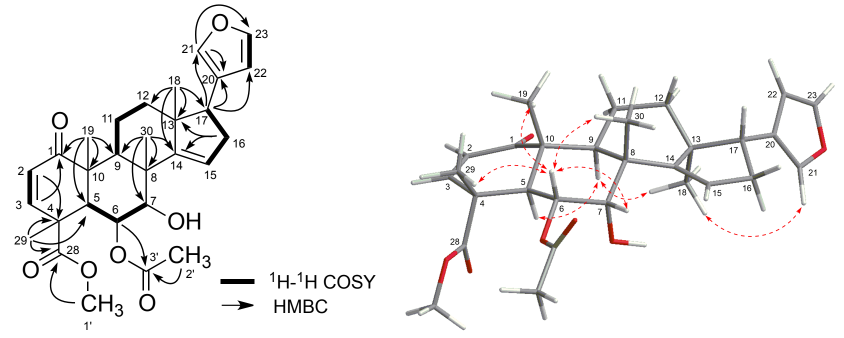

Figure S5. Selected 2D NMR correlations of **6**.

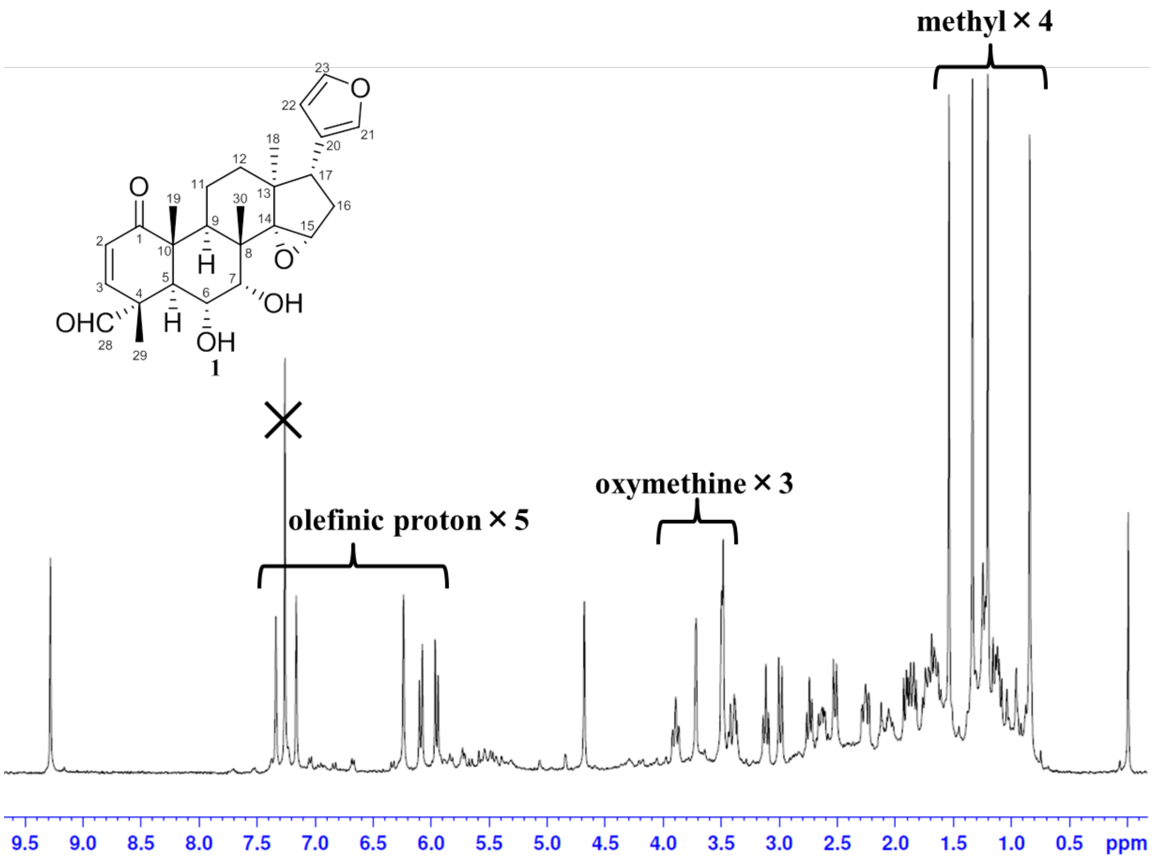

Figure S6. ^{1}H NMR spectrum of ceramicine U (**1**) in $CDCl_3$.

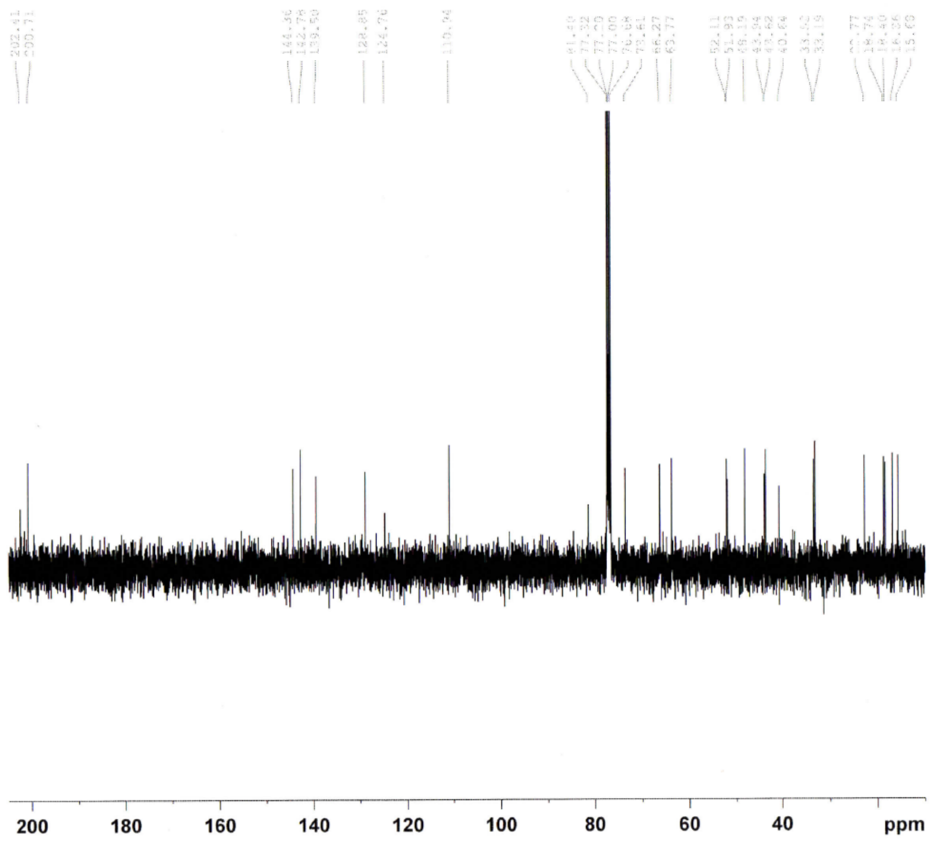

Figure S7. ^{13}C NMR spectrum of ceramicine U (**1**) in $CDCl_3$.

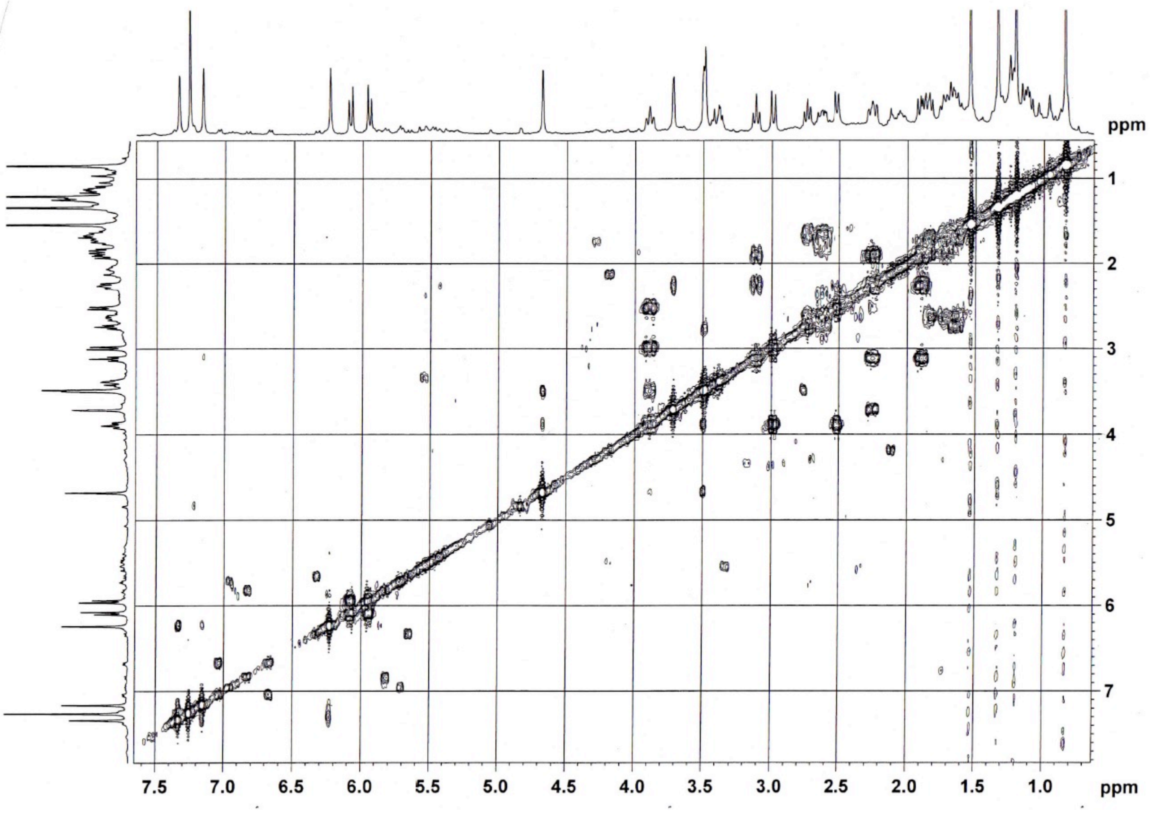

Figure S8. ^{1}H-^{1}H COSY spectrum of ceramicine U (**1**) in $CDCl_3$.

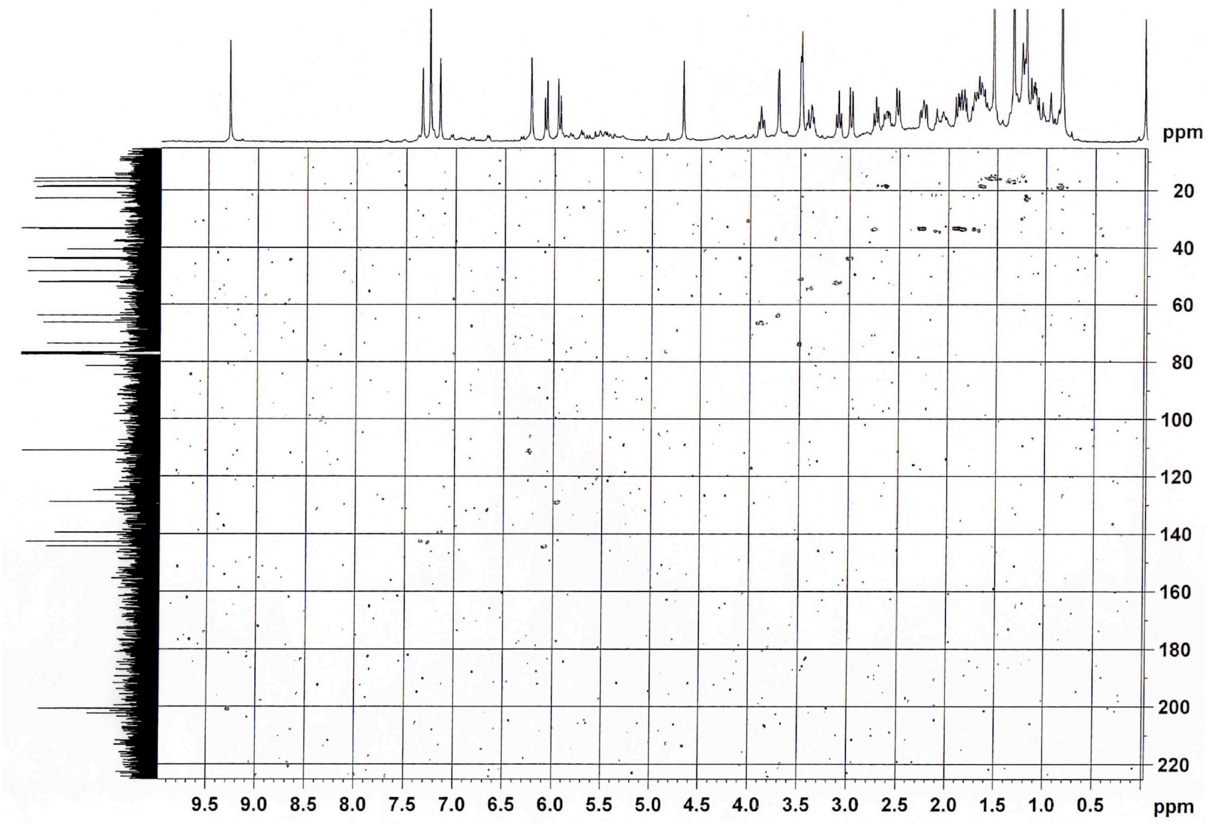

Figure S9. HSQC spectrum of ceramicine U (**1**) in $CDCl_3$.

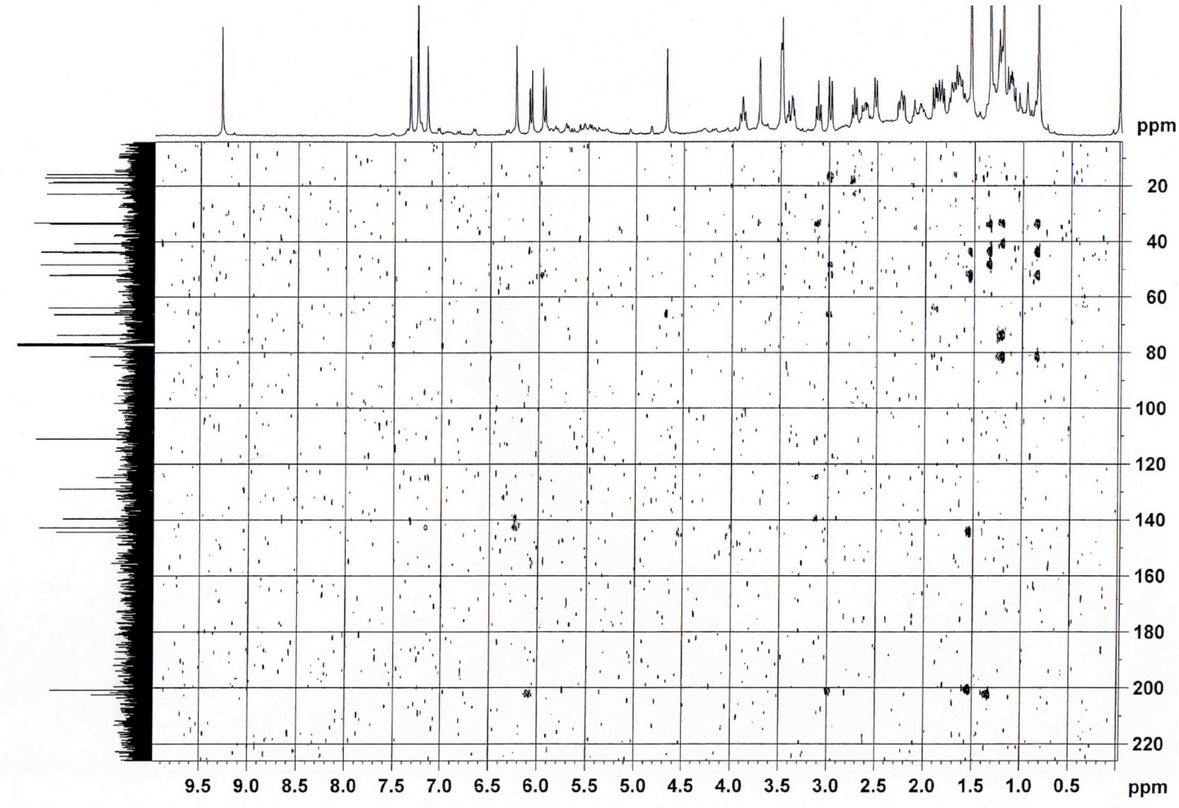

Figure S10. HMBC spectrum of ceramicine U (**1**) in $CDCl_3$.

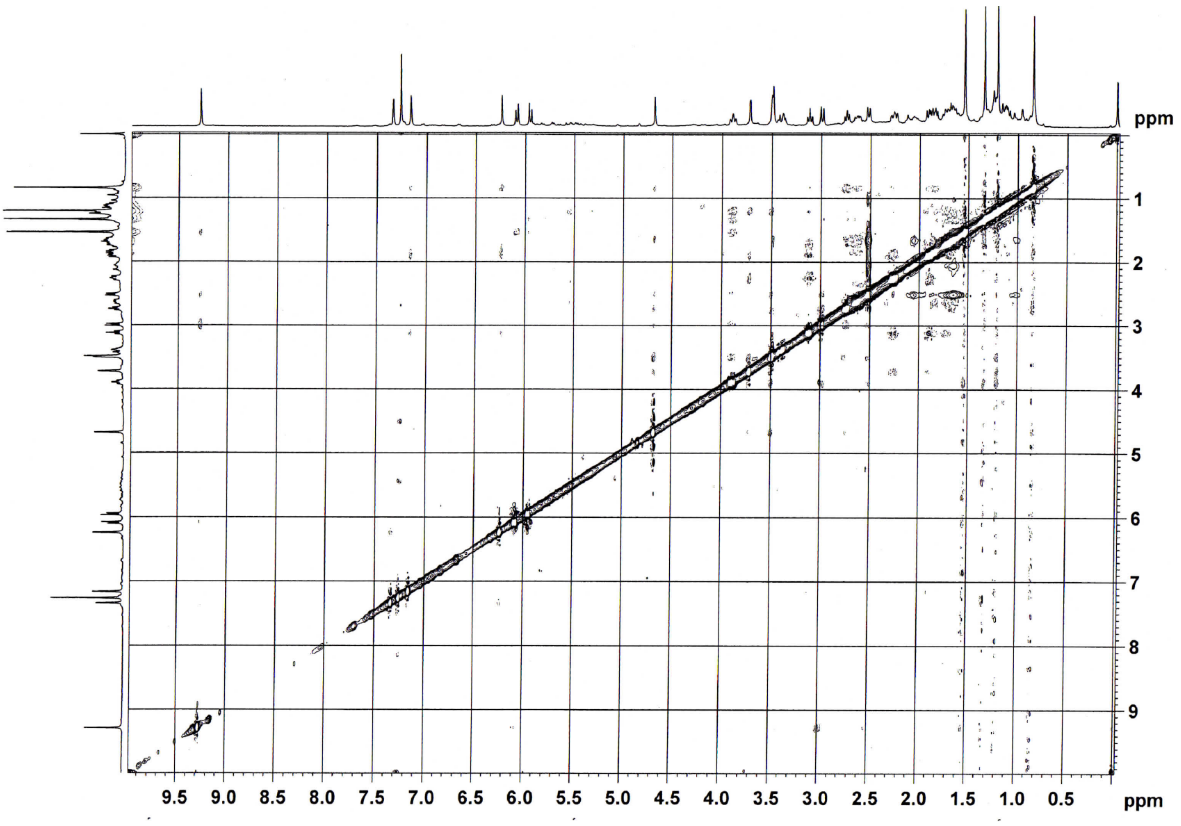

Figure S11. NOESY spectrum of ceramicine U (**1**) in $CDCl_3$.

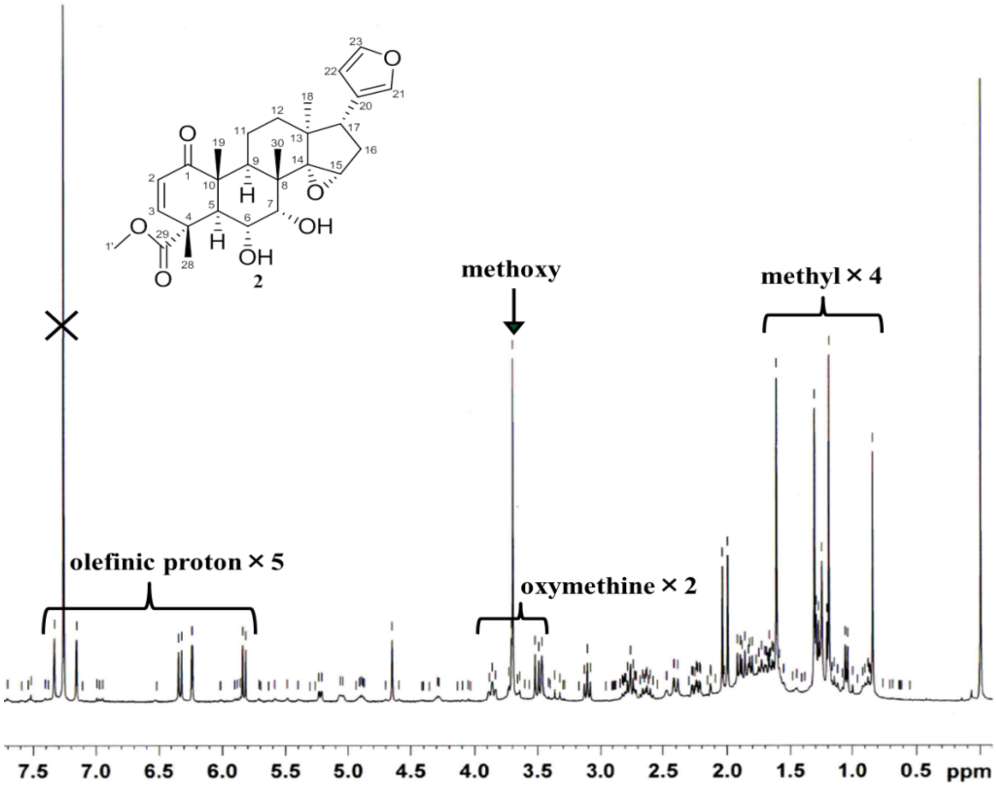

Figure S12. ^{1}H NMR spectrum of ceramicine V (**2**) in $CDCl_3$.

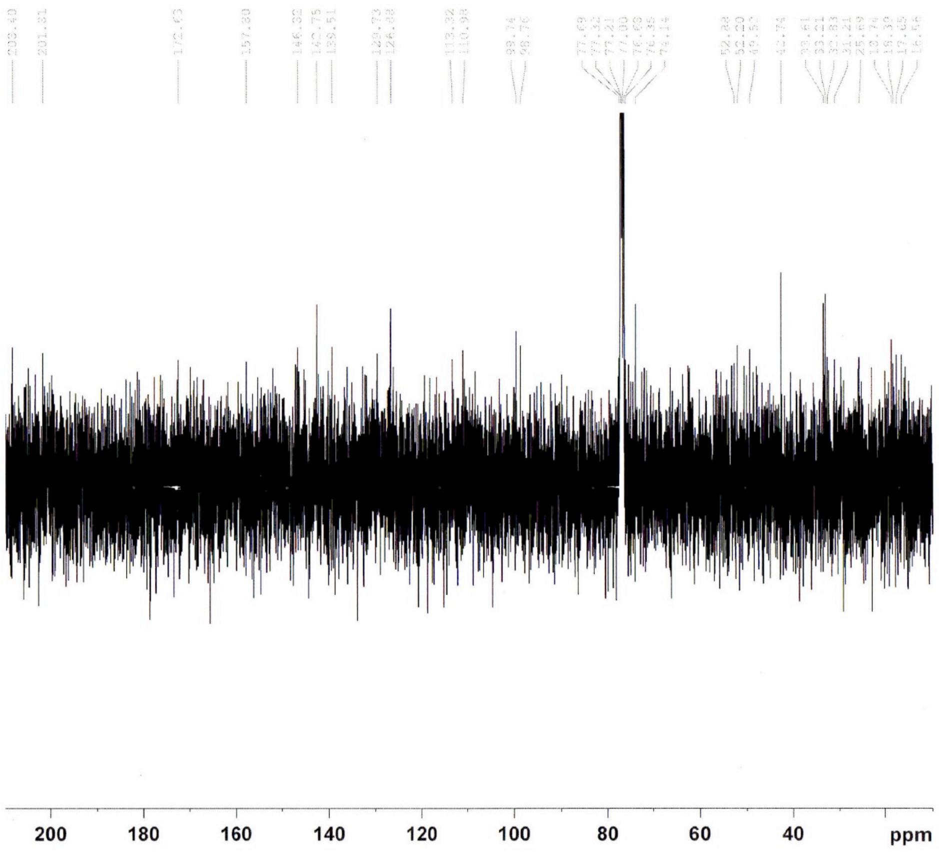

Figure S13. ^{13}C NMR spectrum of ceramicine V (**2**) in $CDCl_3$.

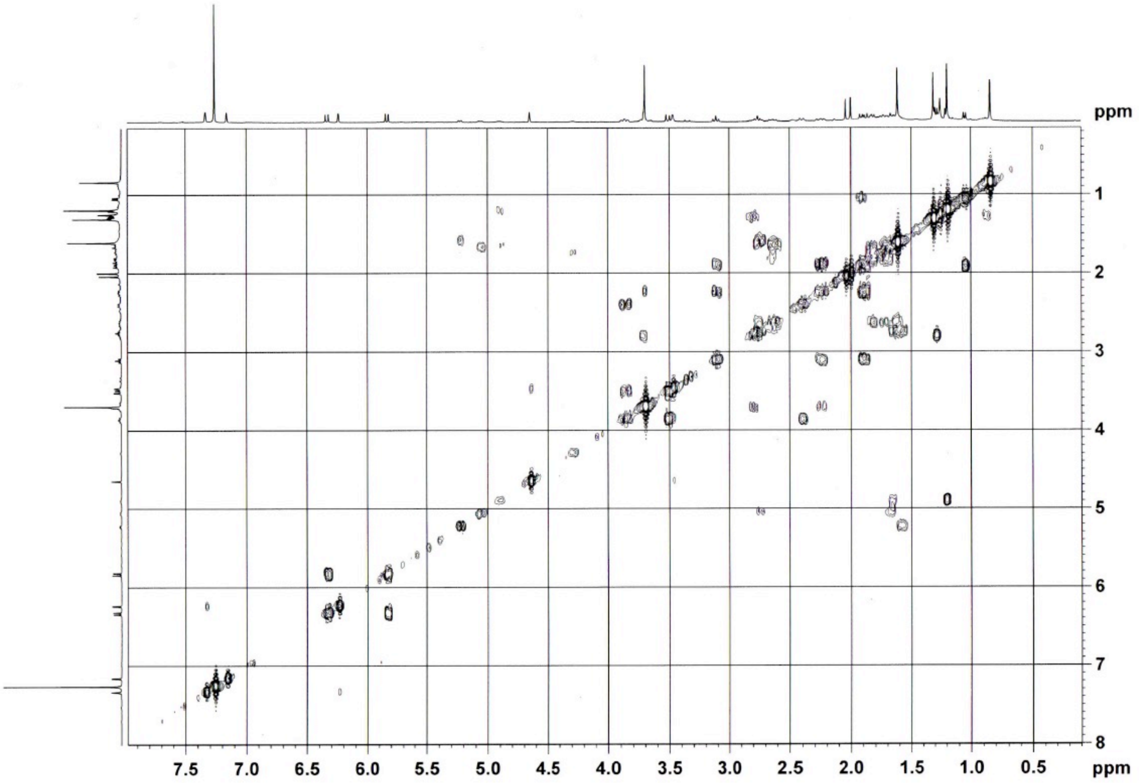

Figure S14. ^1H-^1H COSY spectrum of ceramicine V (**2**) in $CDCl_3$.

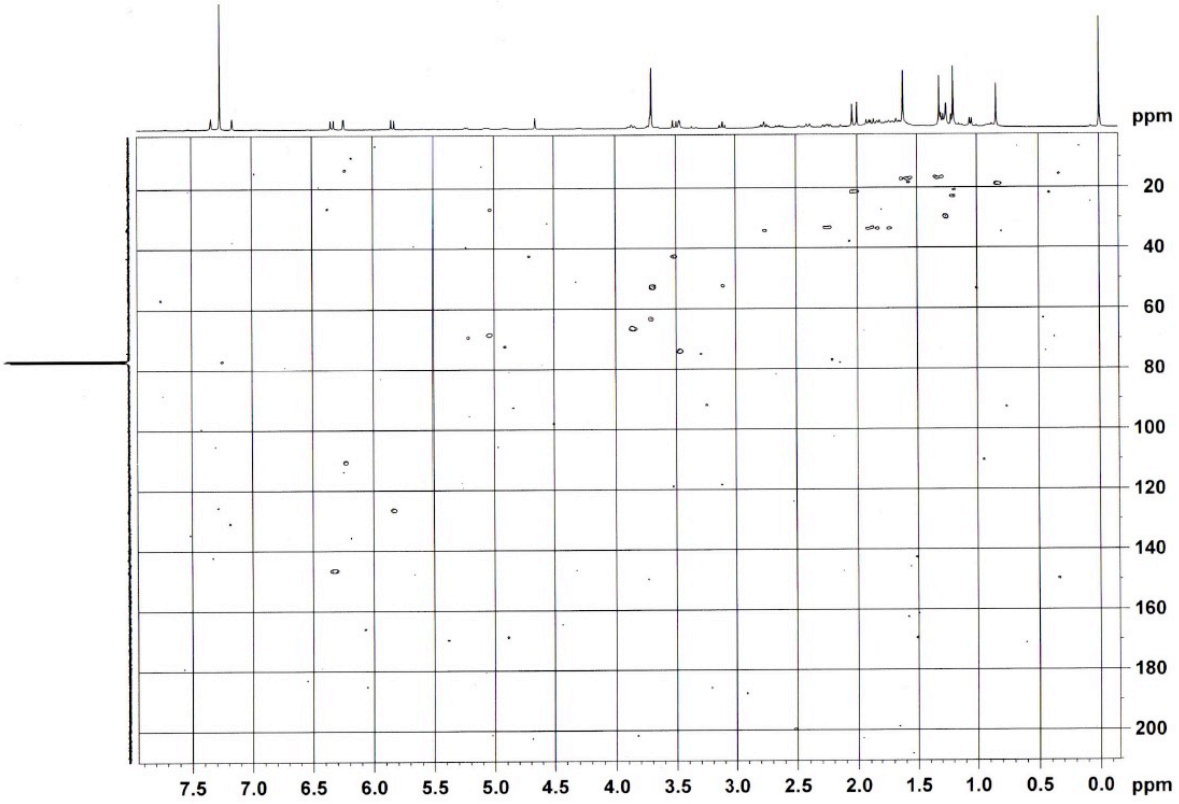

Figure S15. HSQC spectrum of ceramicine V (**2**) in $CDCl_3$.

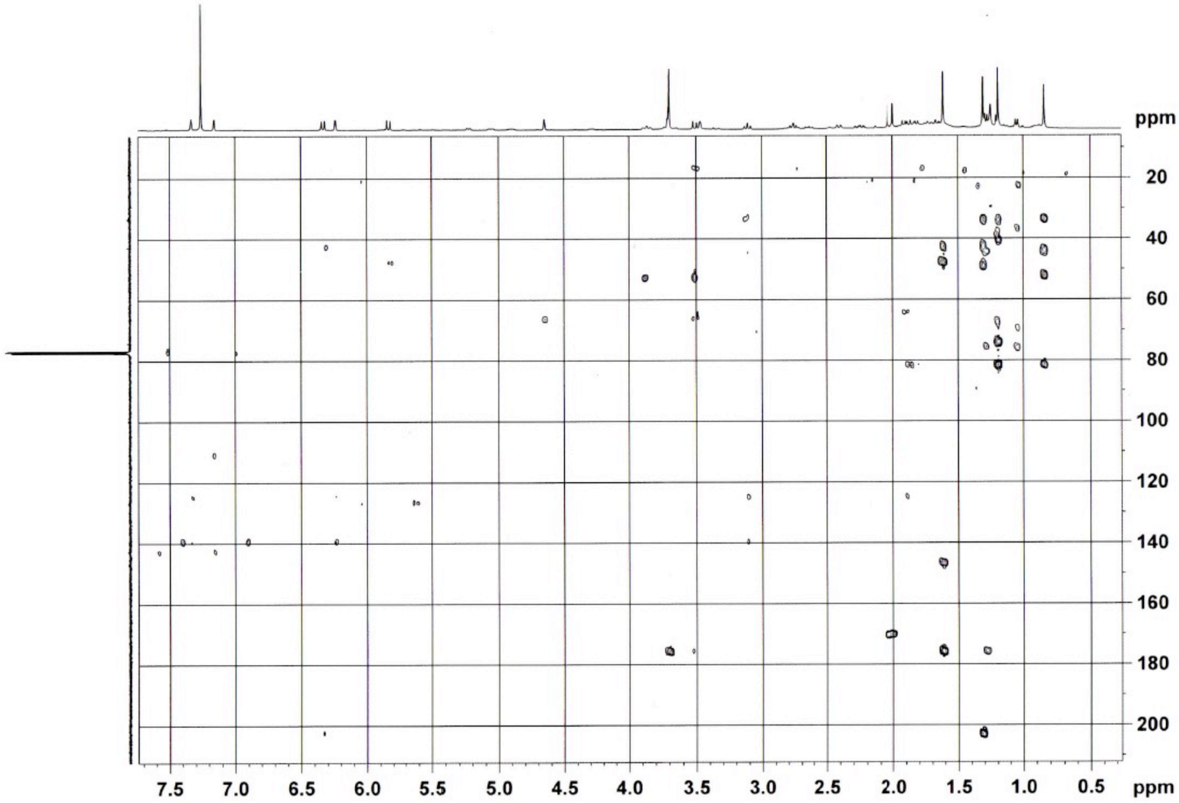

Figure S16. HMBC spectrum of ceramicine V (**2**) in $CDCl_3$.

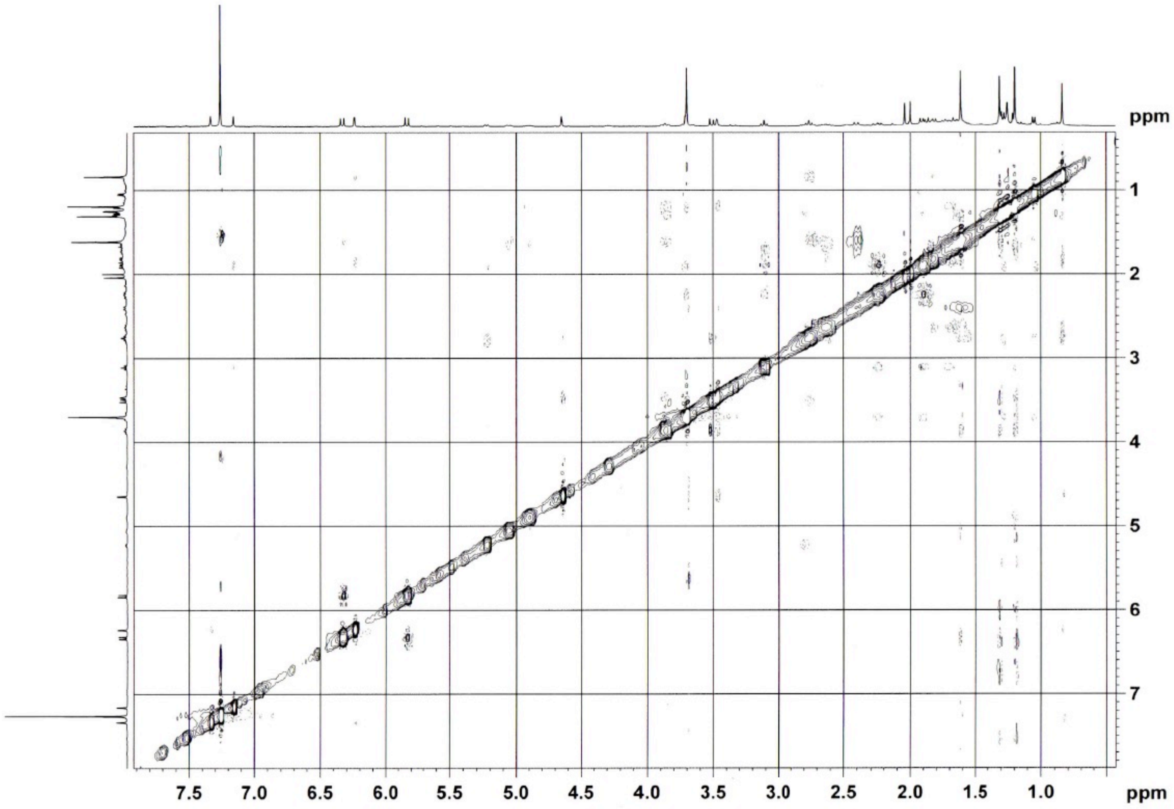

Figure S17. NOESY spectrum of ceramicine V (**2**) in $CDCl_3$.

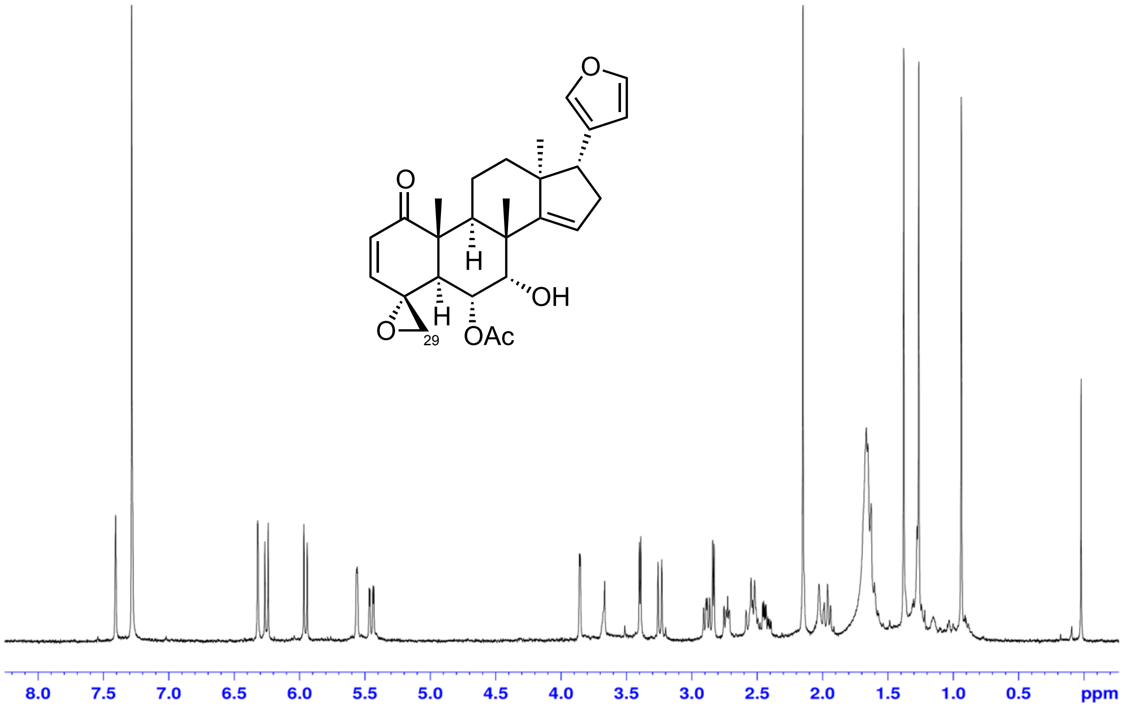

Figure S18. ^{1}H NMR spectrum of ceramicine W (**3**) in $CDCl_3$.

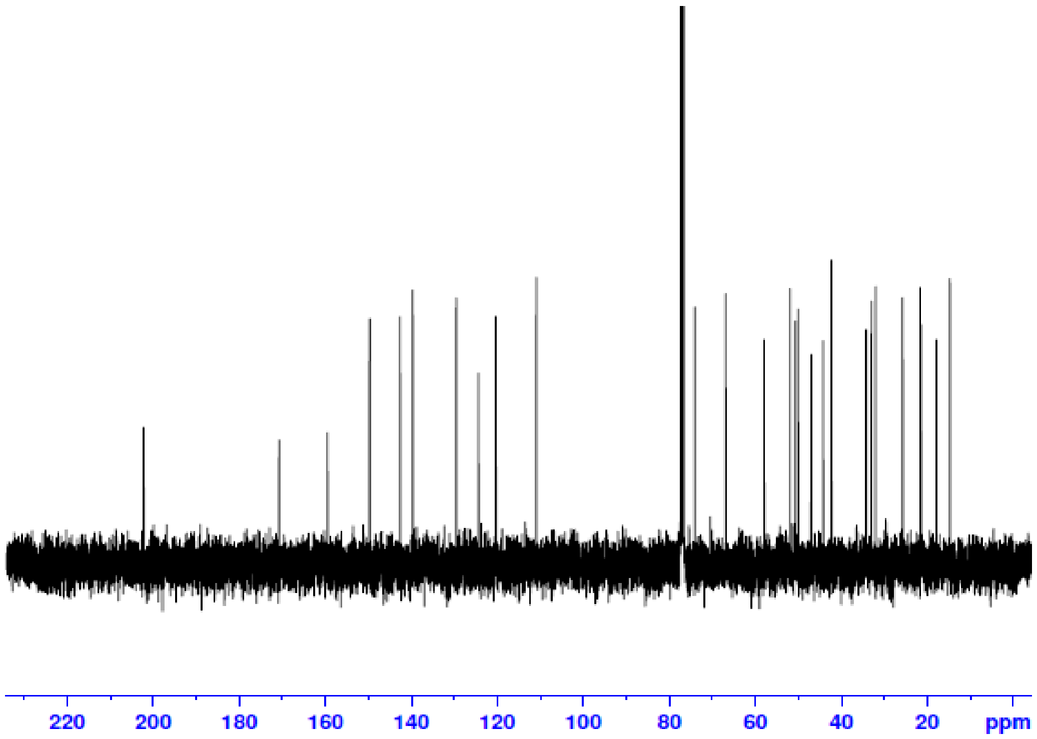

Figure S19. ^{13}C NMR spectrum of ceramicine W (**3**) in $CDCl_3$.

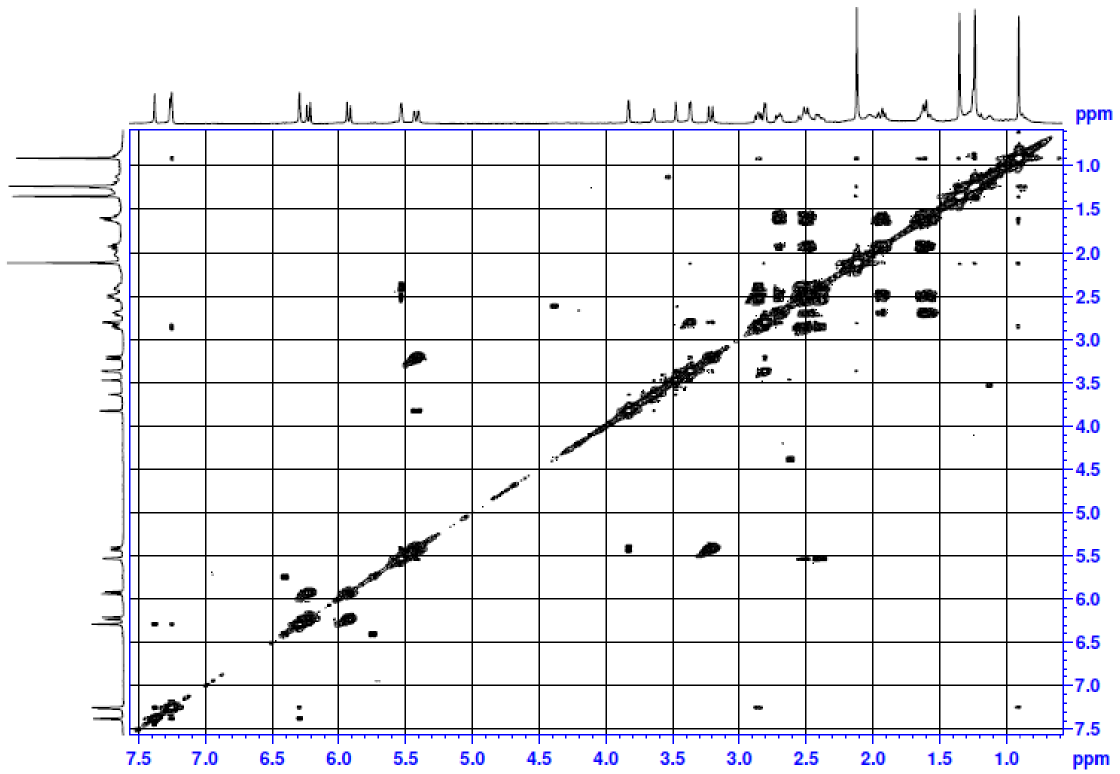

Figure S20. ^{1}H-^{1}H COSY spectrum of ceramicine W (**3**) in $CDCl_3$.

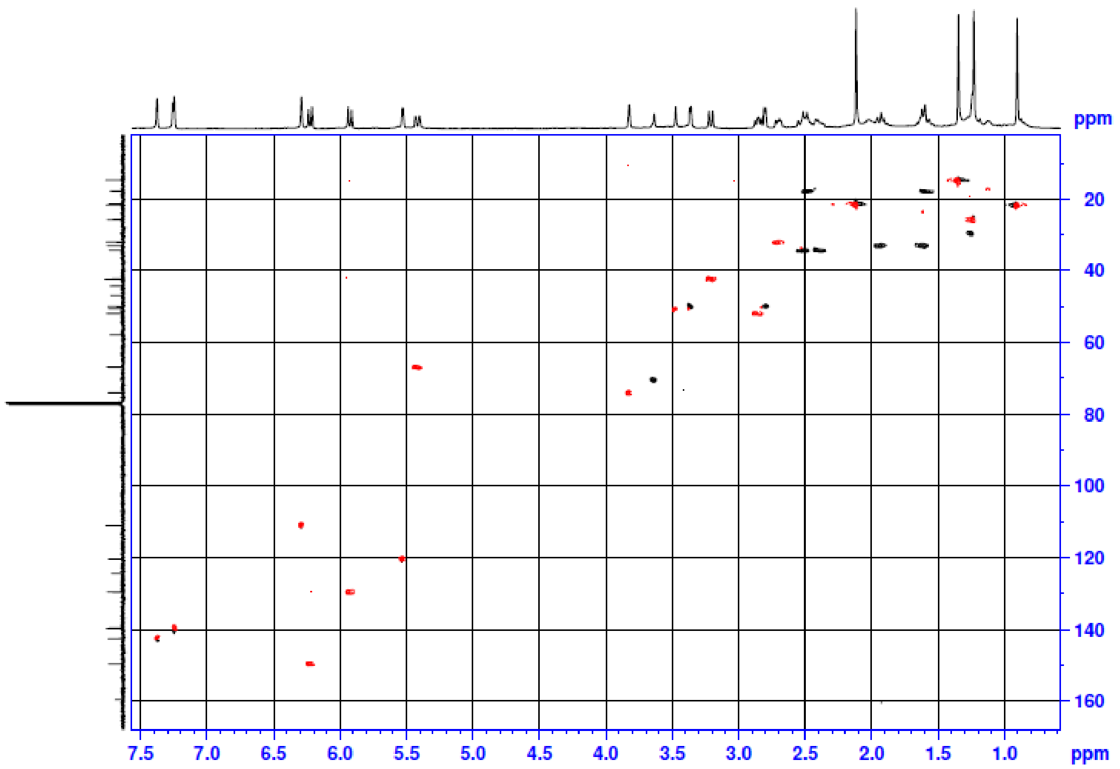

Figure S21. HSQC spectrum of ceramicine W (**3**) in $CDCl_3$.

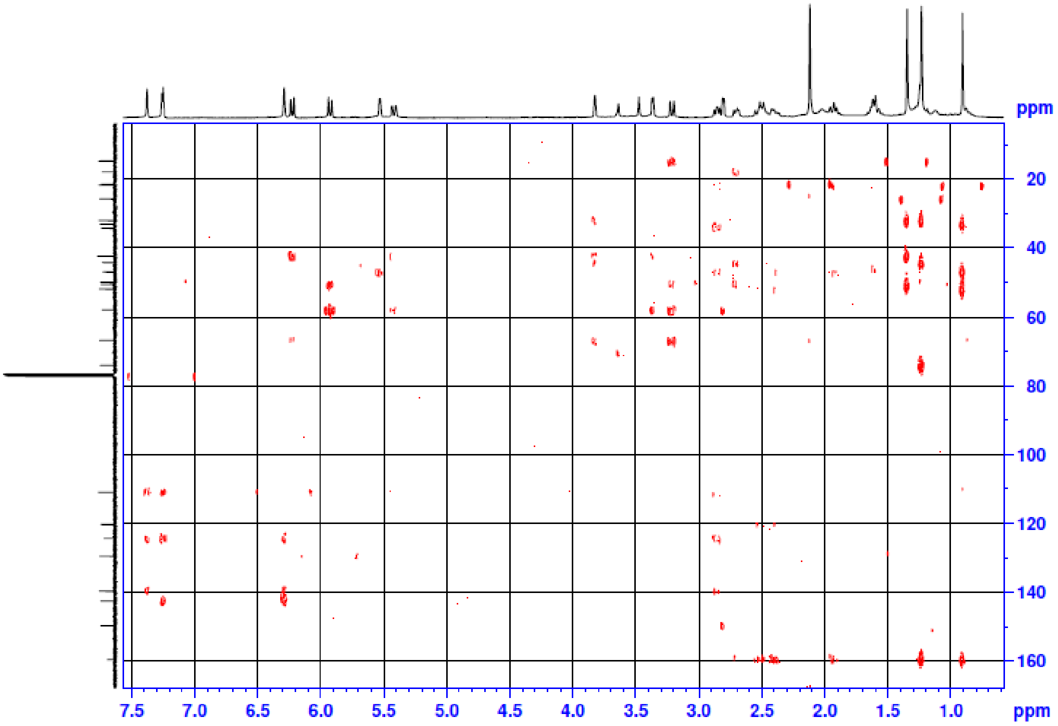

Figure S22. HMBC spectrum of ceramicine W (**3**) in $CDCl_3$.

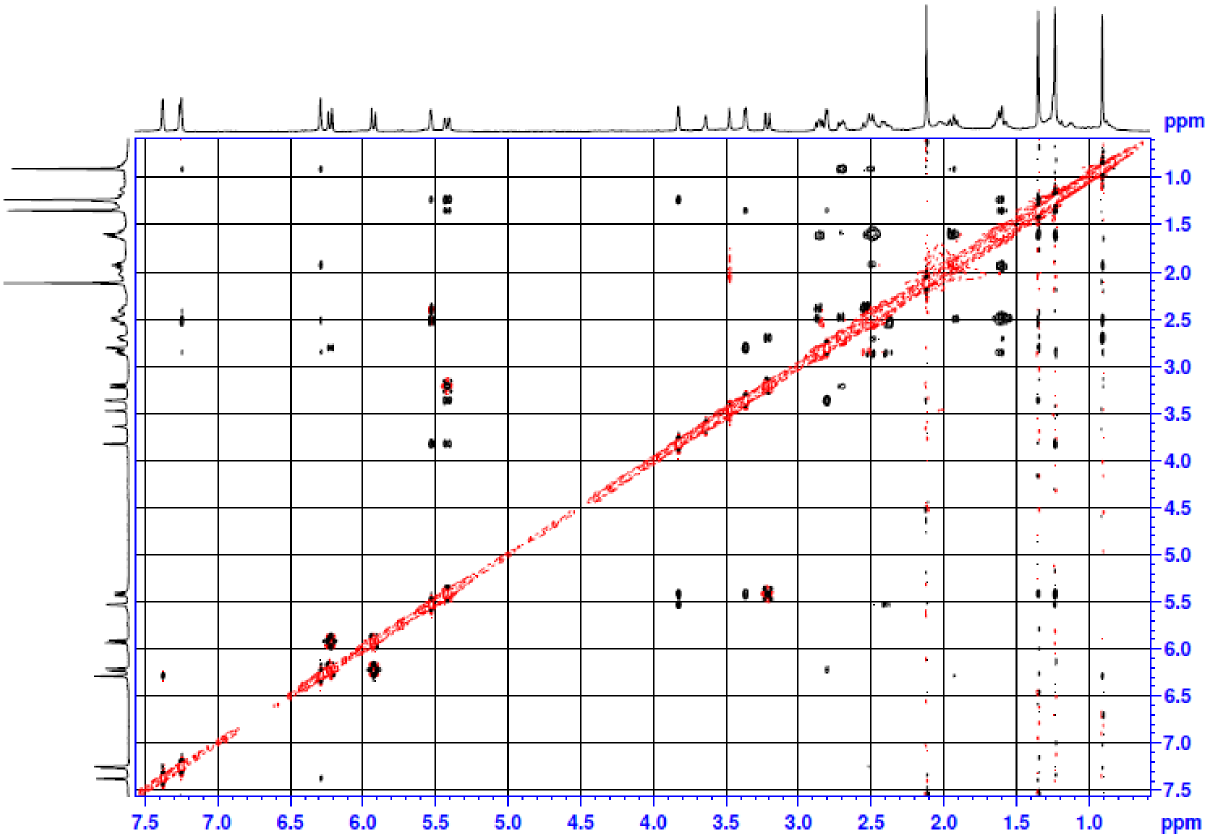

Figure S23. NOESY spectrum of ceramicine W (**3**) in $CDCl_3$.

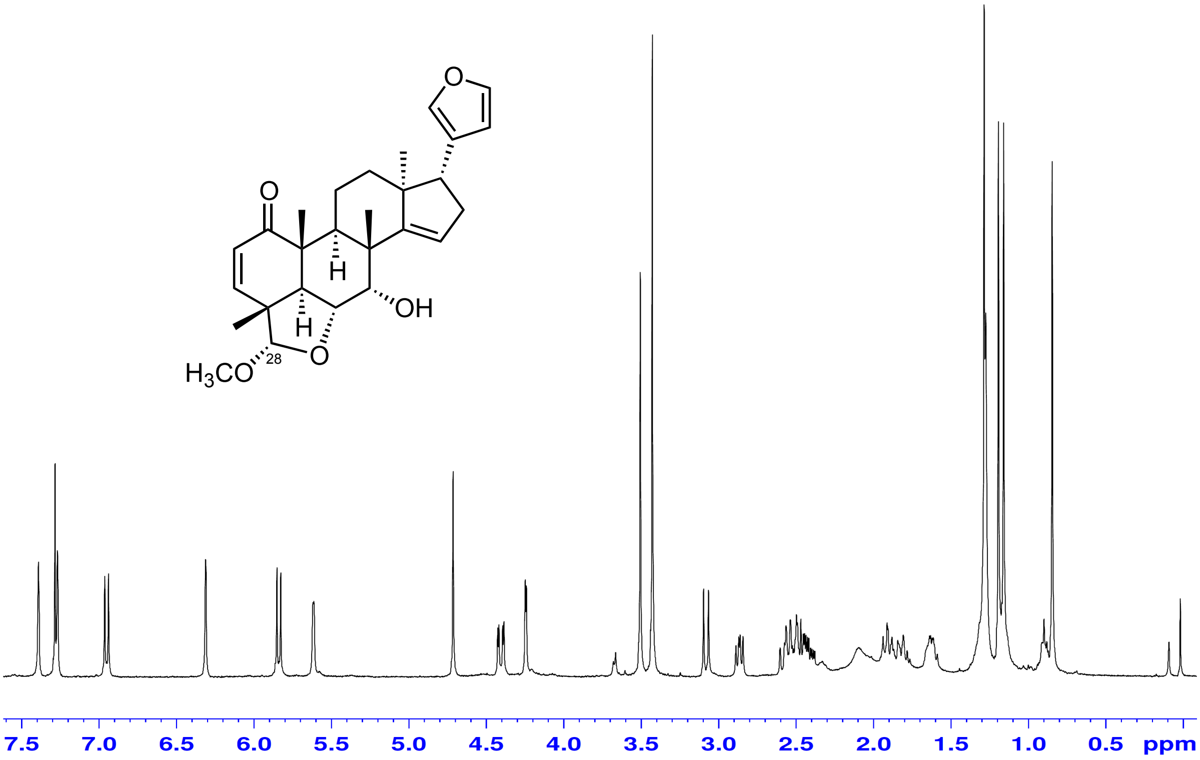

Figure S24. ^{1}H NMR spectrum of ceramicine X (**4**) in $CDCl_3$.

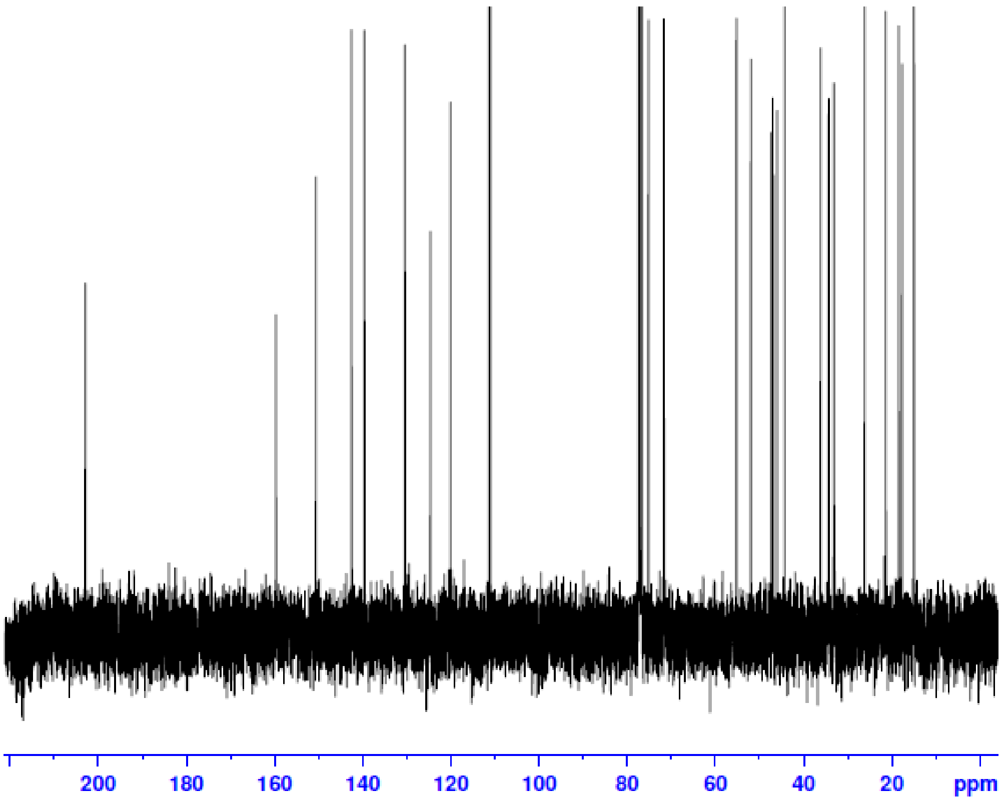

Figure S25. ^{13}C NMR spectrum of ceramicine X (**4**) in $CDCl_3$.

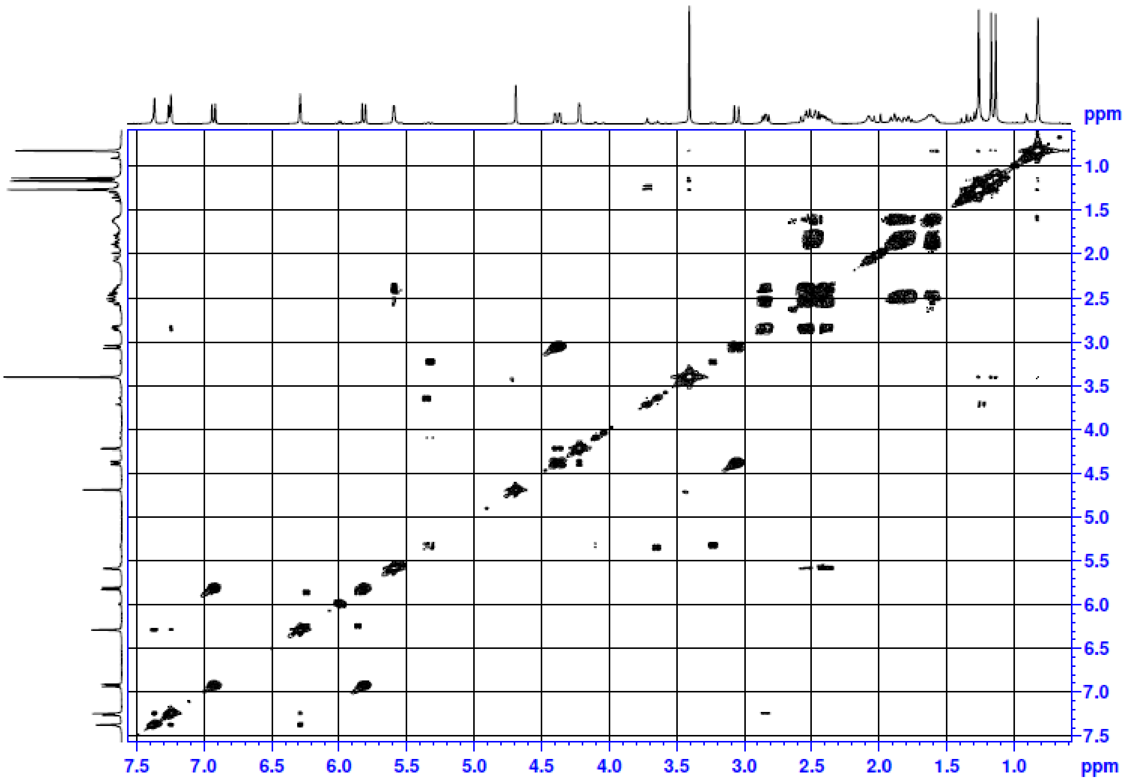

Figure S26. ^{1}H-^{1}H COSY spectrum of ceramicine X (**4**) in $CDCl_3$.

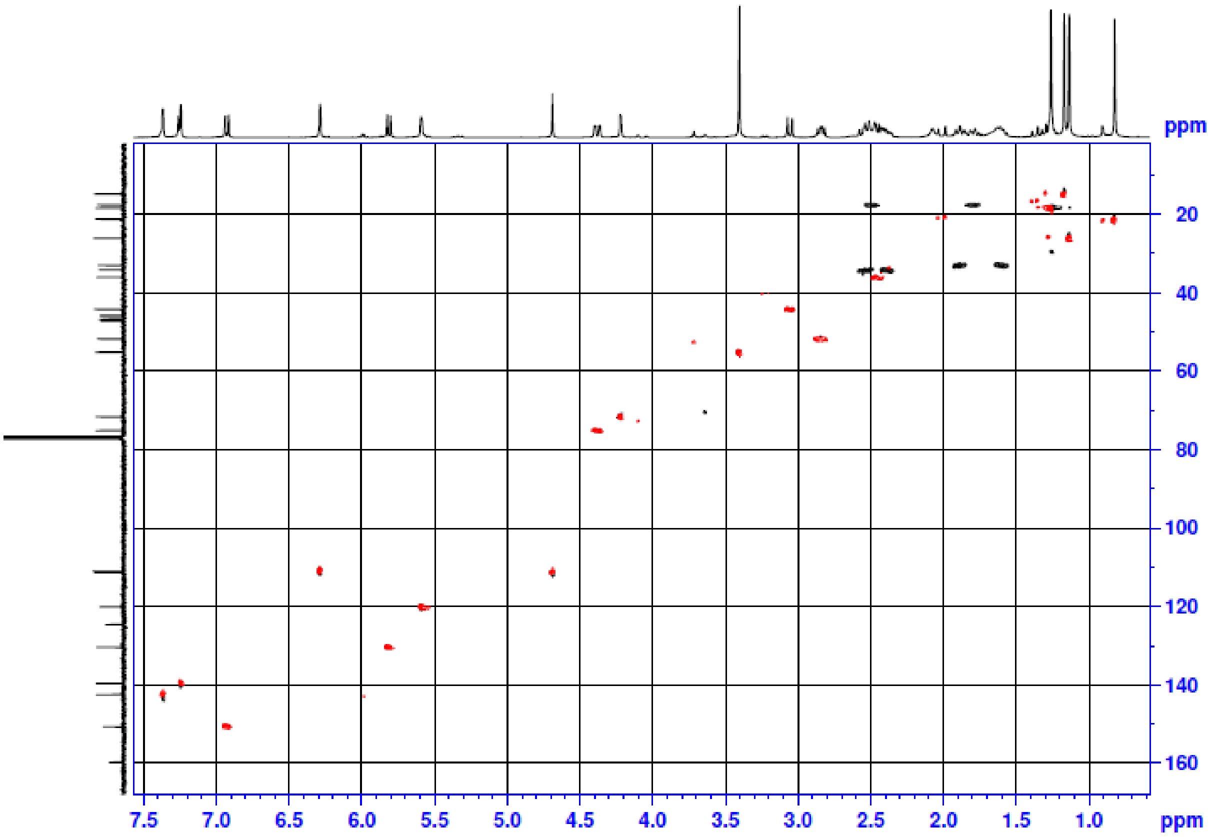

Figure S27. HSQC spectrum of ceramicine X (**4**) in $CDCl_3$.

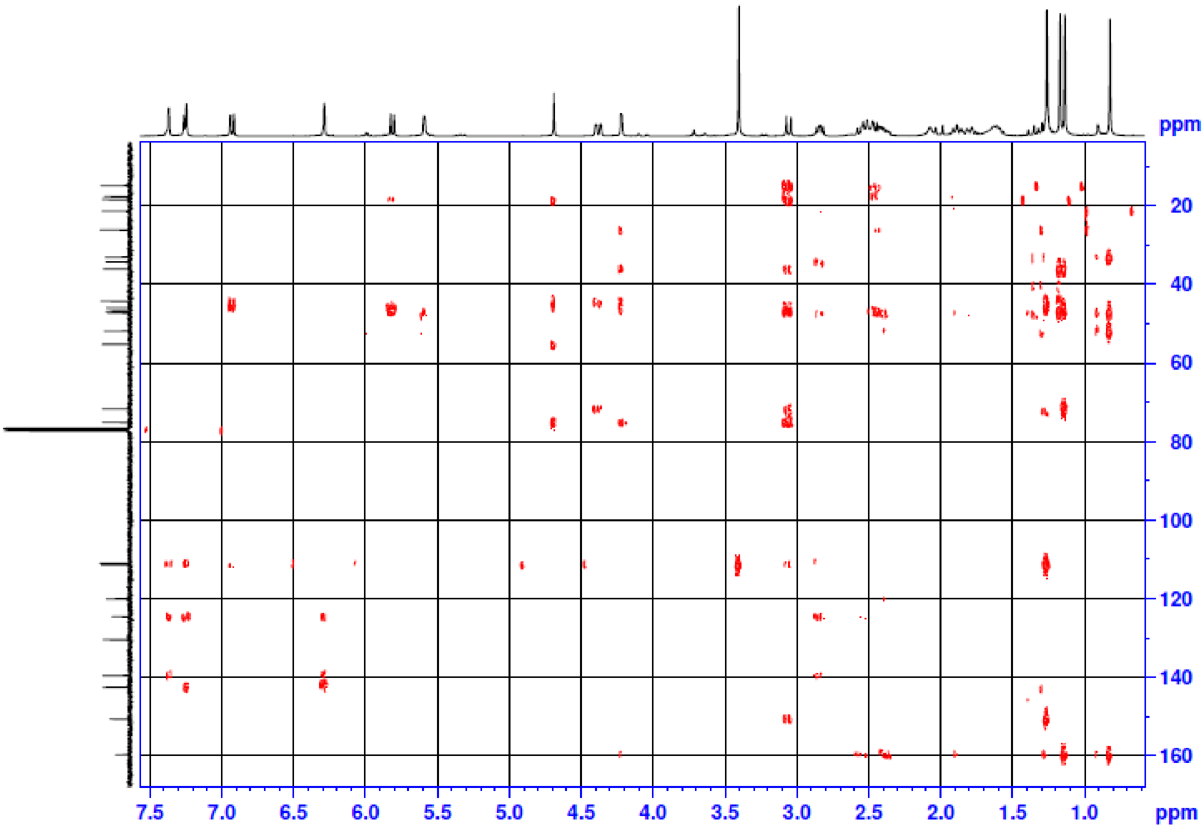

Figure S28. HMBC spectrum of ceramicine X (**4**) in $CDCl_3$.

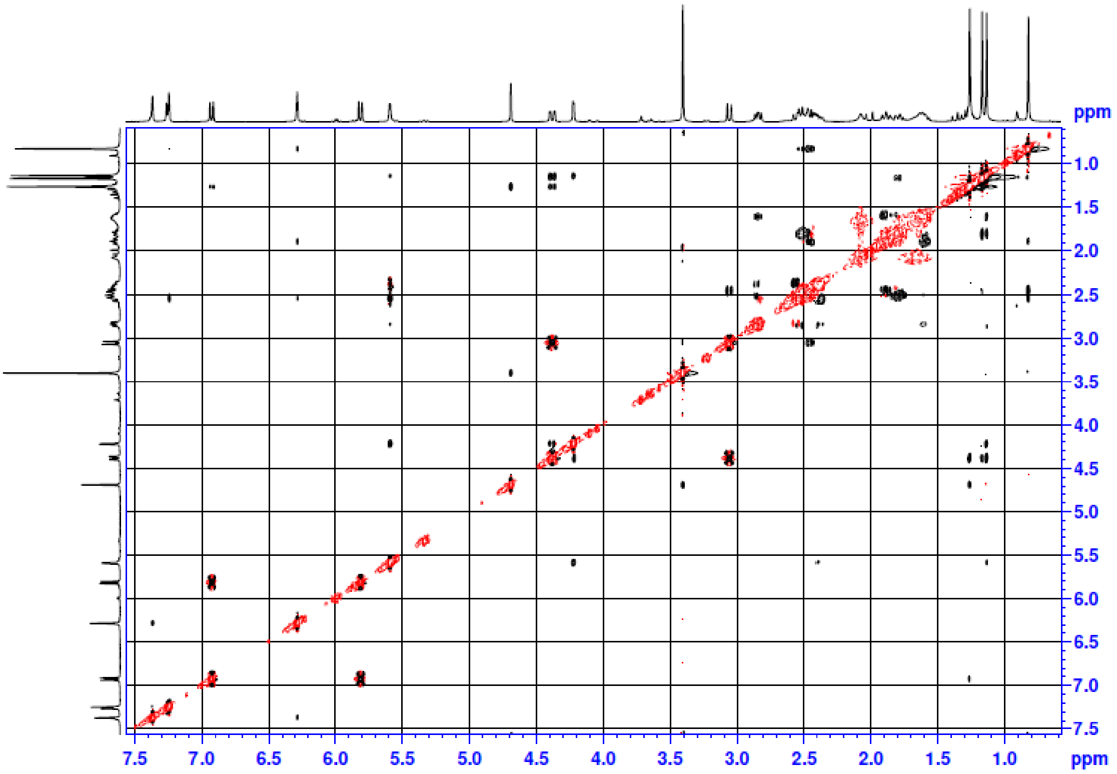

Figure S29. NOESY spectrum of ceramicine X (**4**) in $CDCl_3$.

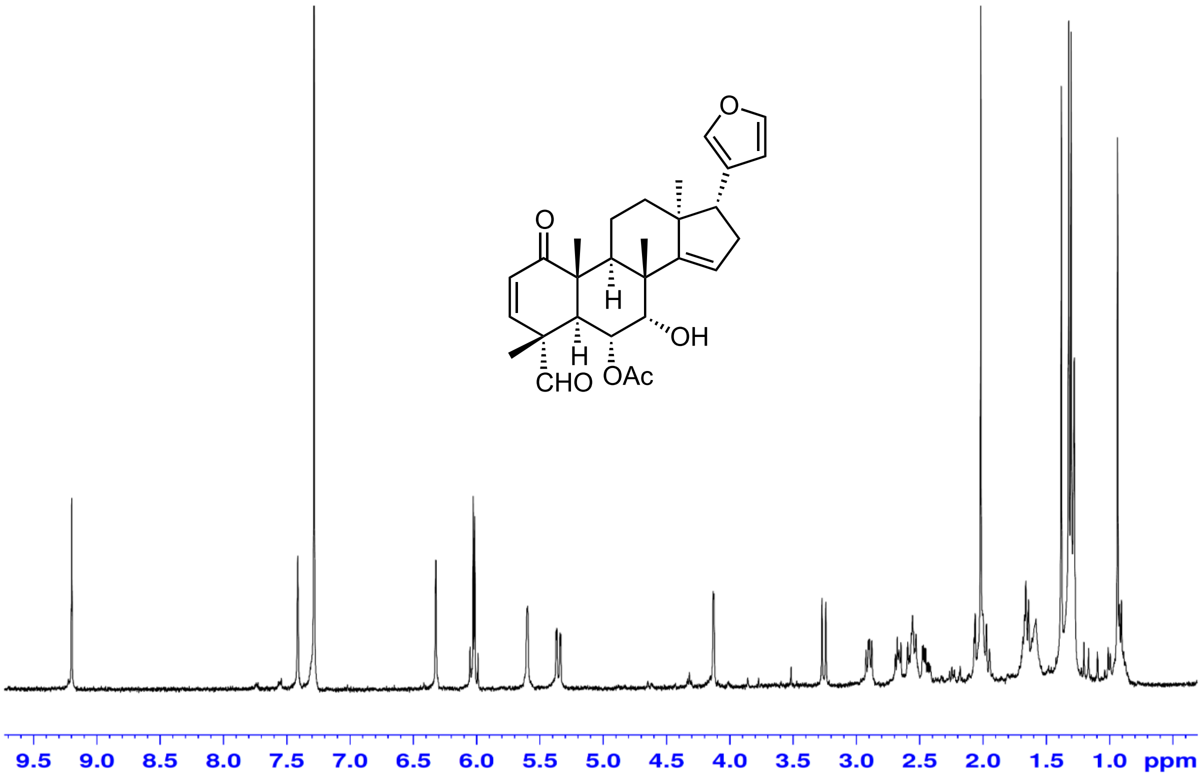

Figure S30. ^{1}H NMR spectrum of ceramicine Y (**5**) in $CDCl_3$.

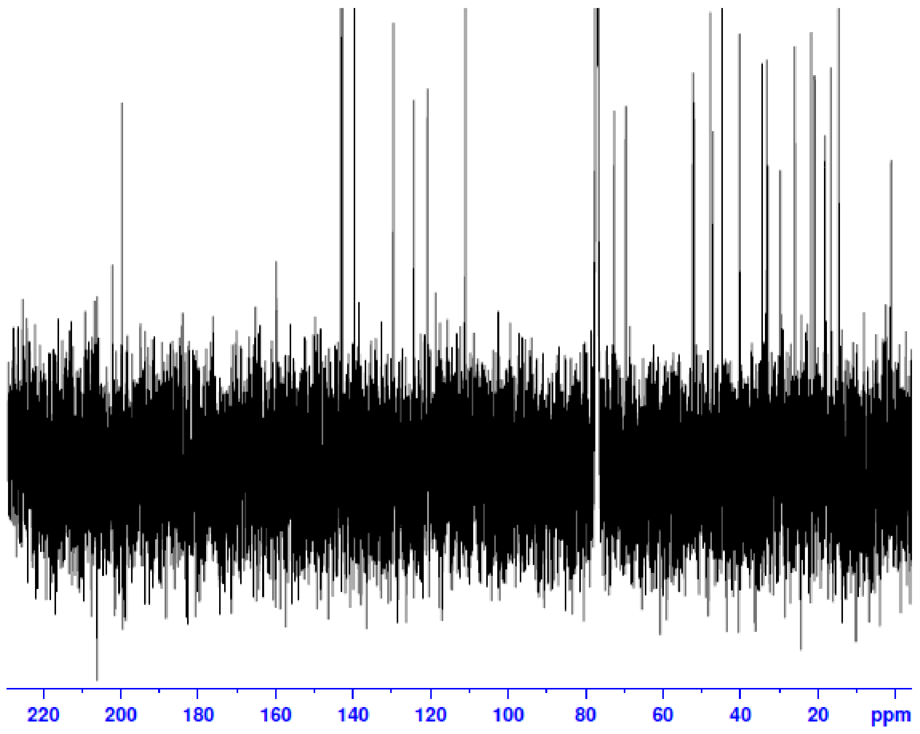

Figure S31. ^{13}C NMR spectrum of ceramicine Y (**5**) in $CDCl_3$.

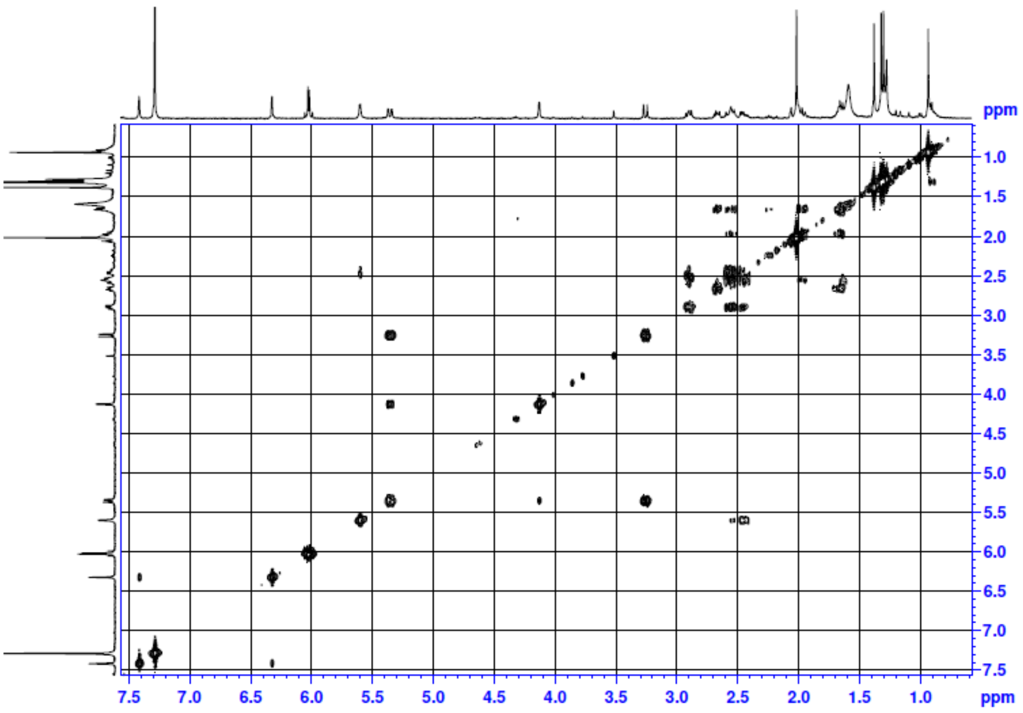

Figure S32. ^{1}H-^{1}H COSY spectrum of ceramicine Y (**5**) in $CDCl_3$.

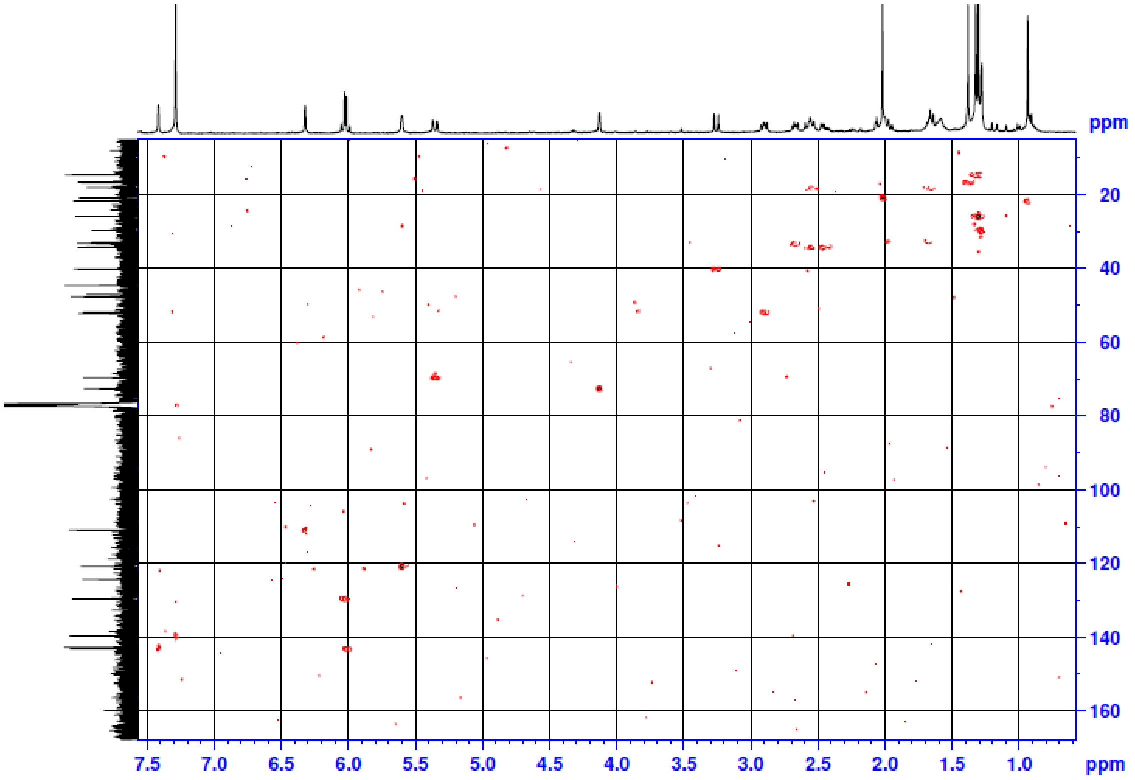

Figure S33. HSQC spectrum of ceramicine Y (**5**) in $CDCl_3$.

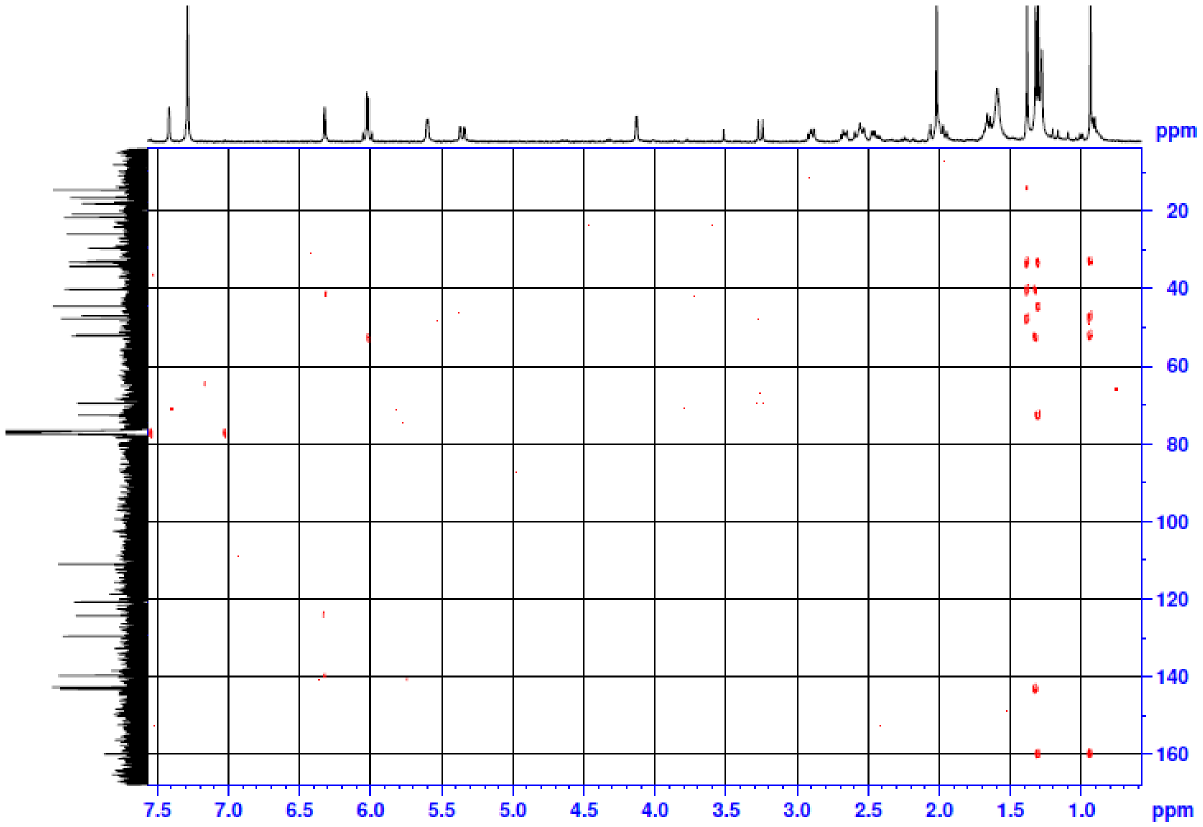

Figure S34. HMBC spectrum of ceramicine Y (**5**) in $CDCl_3$.

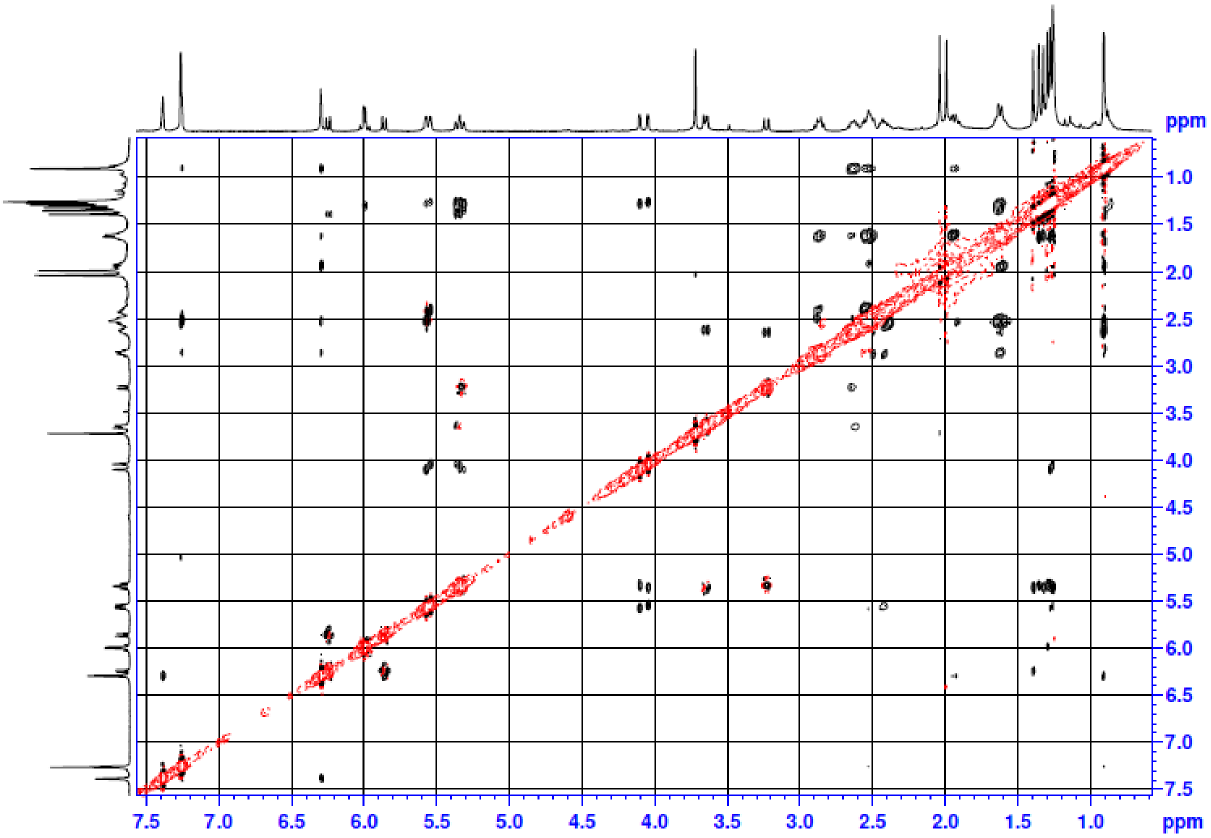

Figure S35. NOESY spectrum of ceramicine Y (**5**) in $CDCl_3$.

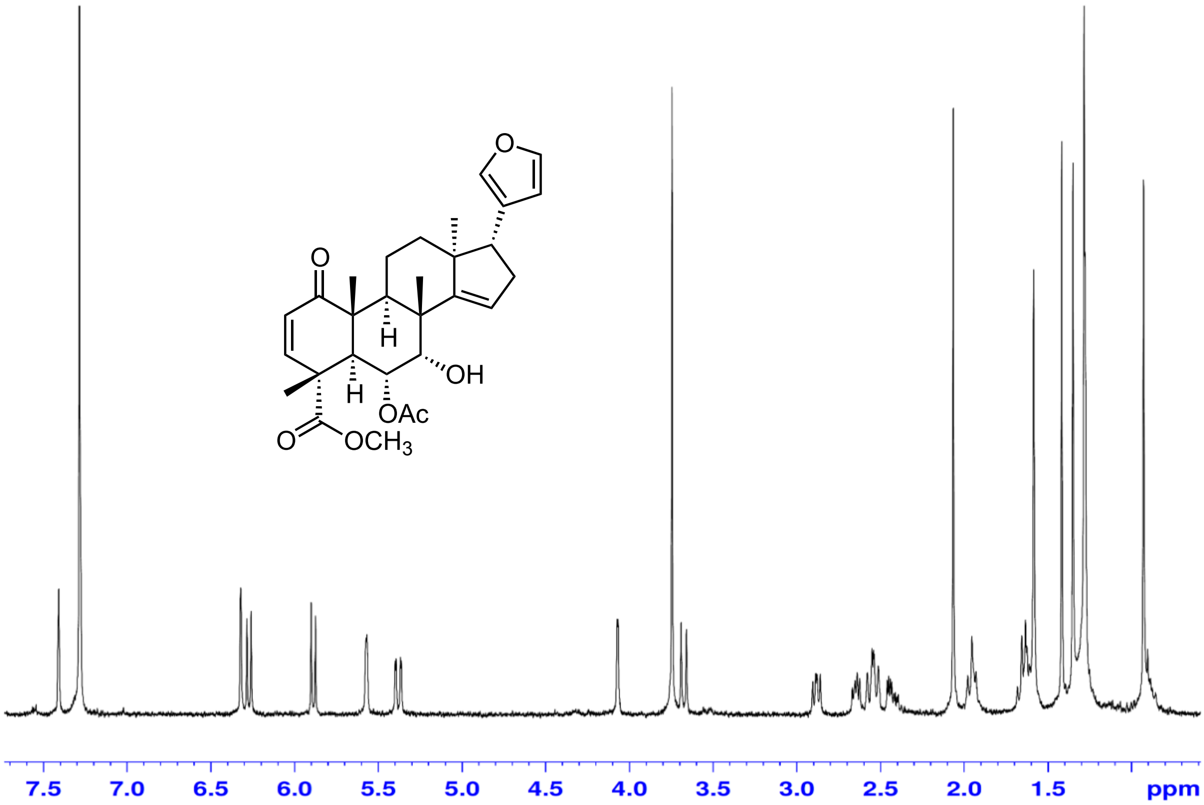

Figure S36. ^{1}H NMR spectrum of ceramicine Z (**6**) in $CDCl_3$.

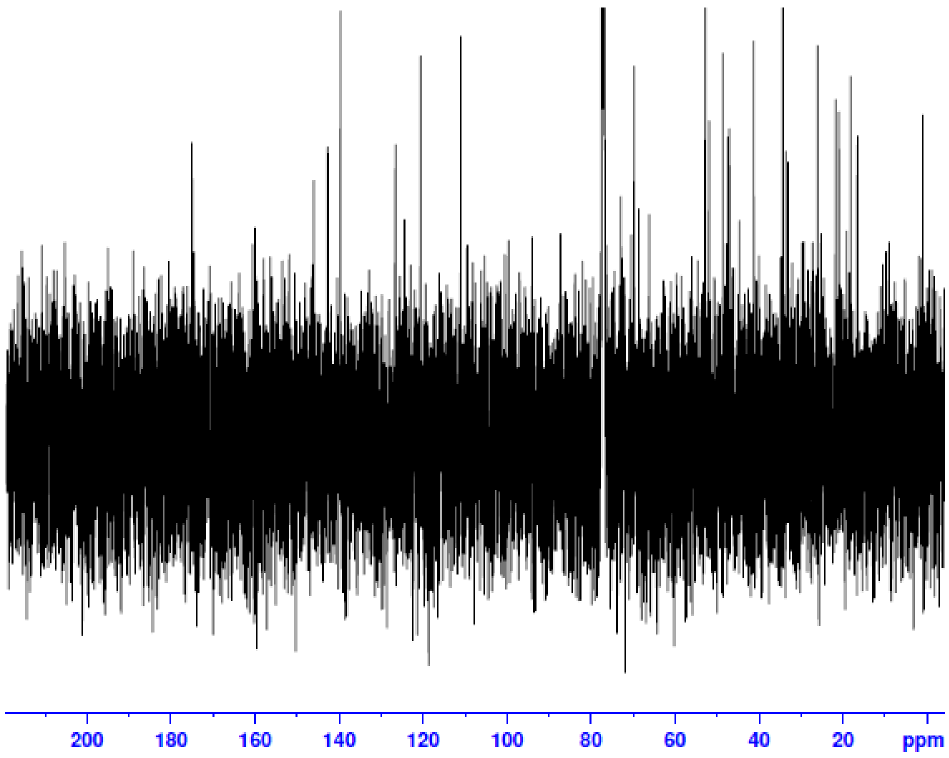

Figure S37. ^{13}C NMR spectrum of ceramicine Z (**6**) in $CDCl_3$.

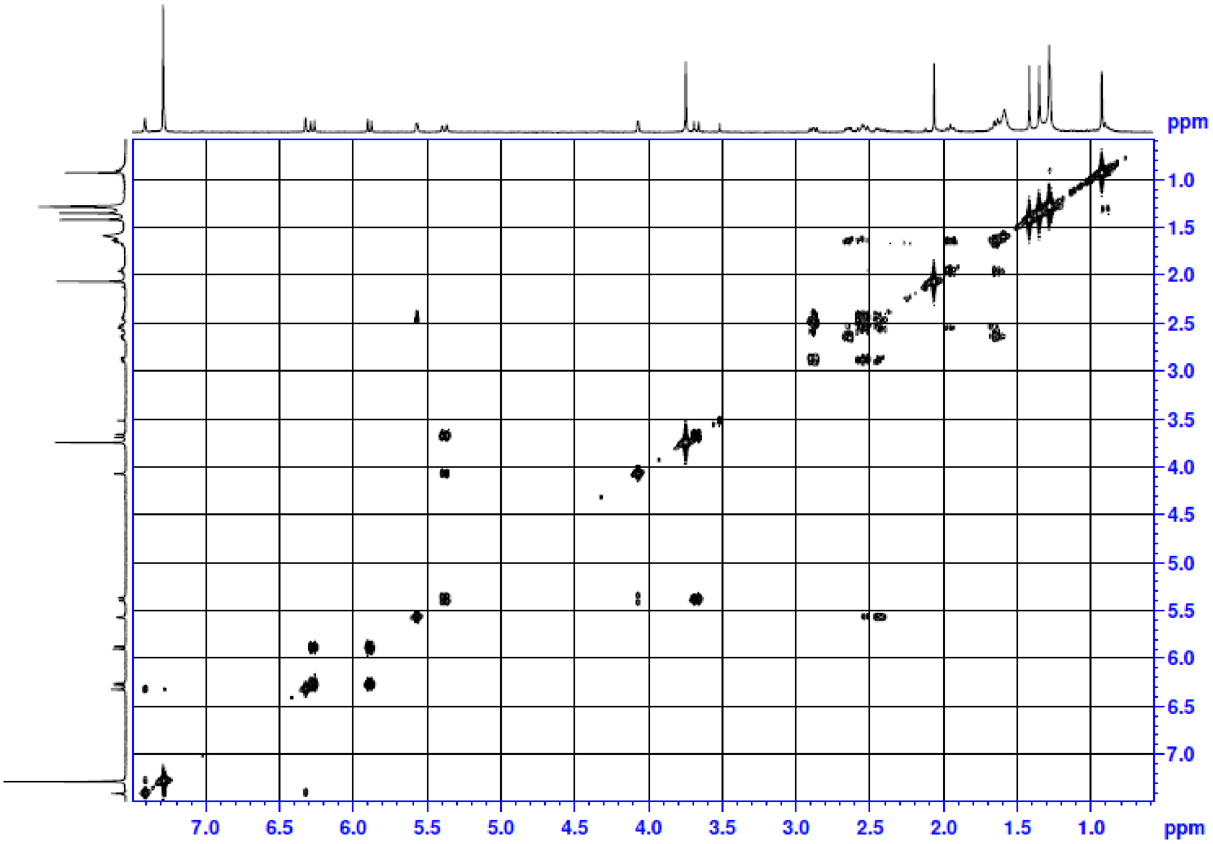

Figure S38. ^{1}H-^{1}H COSY spectrum of ceramicine Z (**6**) in $CDCl_3$.

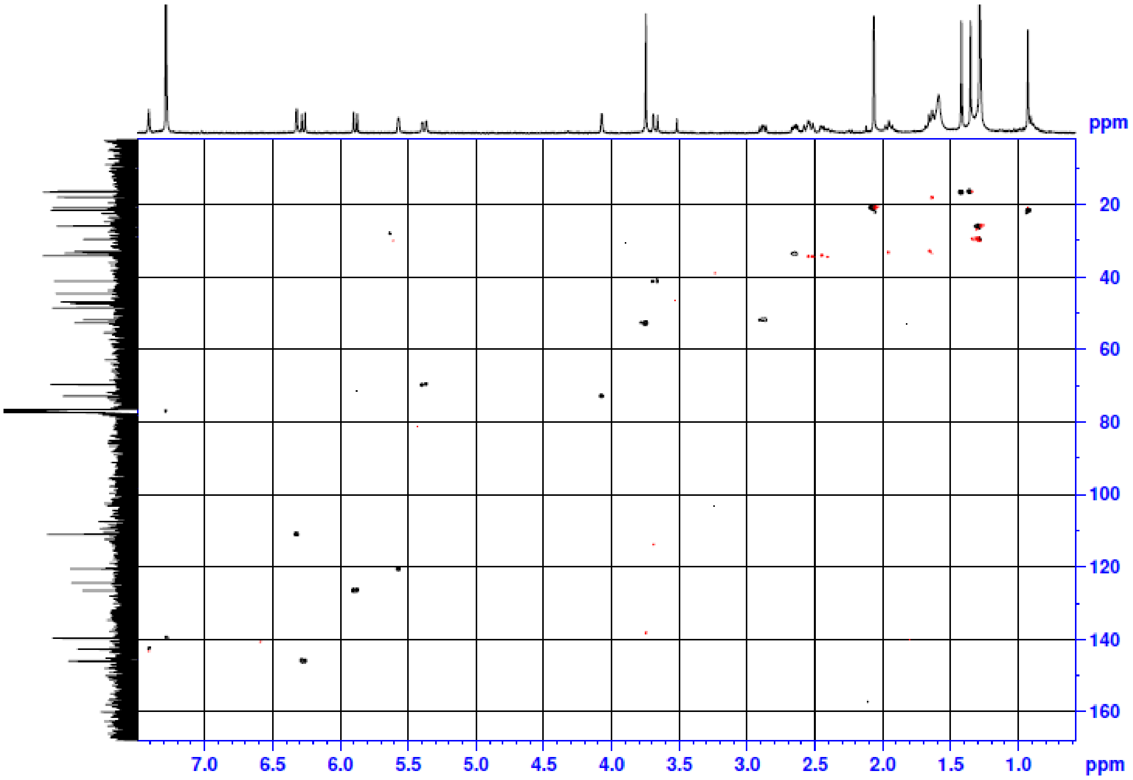

Figure S39. HSQC spectrum of ceramicine Z (**6**) in $CDCl_3$.

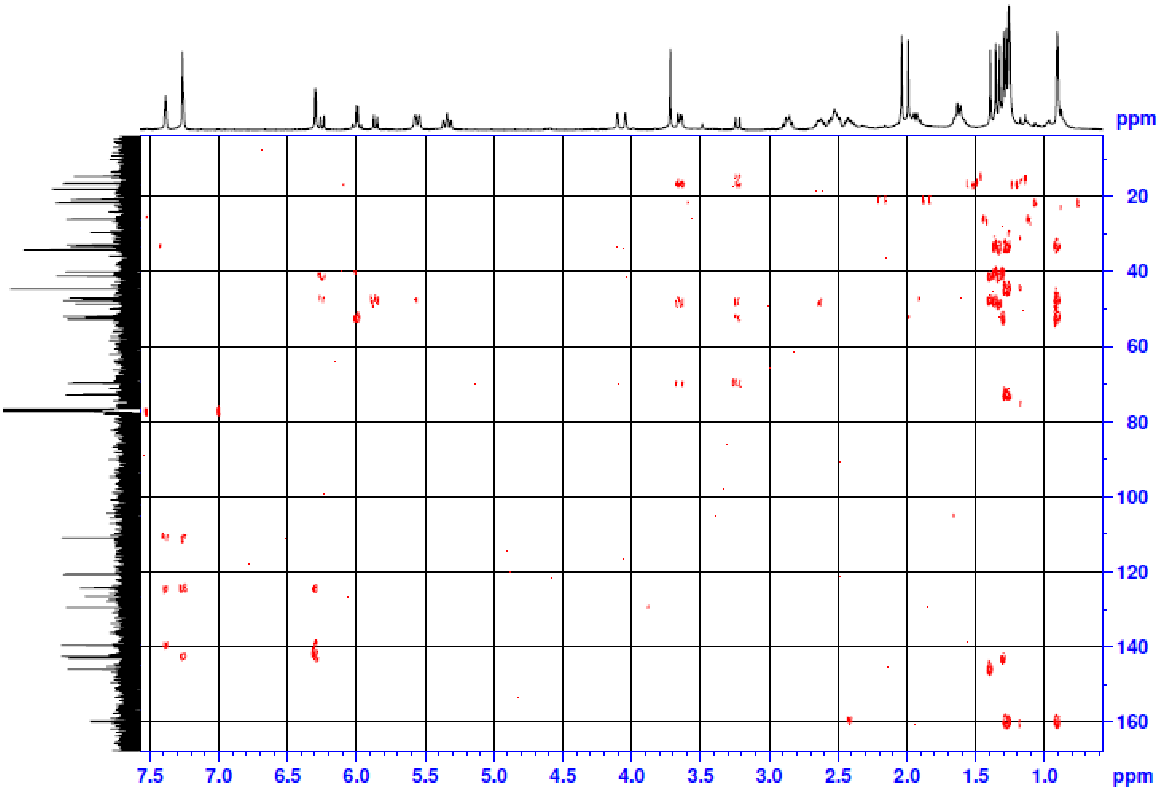

Figure S40. HMBC spectrum of ceramicine Z (**6**) in $CDCl_3$.

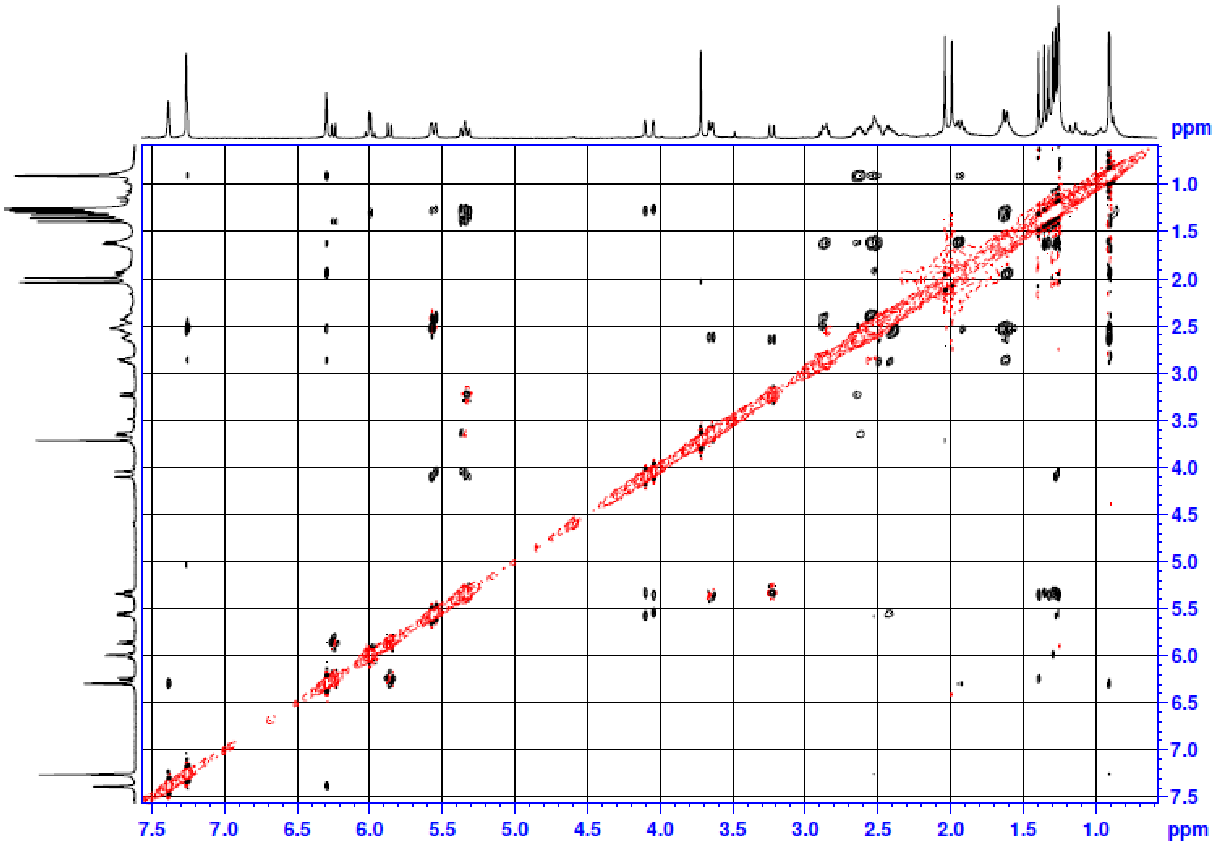

Figure S41. NOESY spectrum of ceramicine Z (**6**) in $CDCl_3$.
